# A Sr–Ga Oxy-Hydroxide with High Thermal Stability: Unraveling Its Characteristic Hydrogen-Bond Network

**DOI:** 10.1021/acs.inorgchem.5c02586

**Published:** 2025-08-31

**Authors:** Yusuke Asai, Yuto Nishihara, Yoko Kokubo, Kenji Arai, Kosaku Ohishi, Satoshi Ogawa, Miwa Saito, Yusuke Nambu, Maxim Avdeev, Koji Kimoto, Zi Lang Goo, Kunihisa Sugimoto, Miki Inada, Katsuro Hayashi, Teruki Motohashi

**Affiliations:** † Department of Applied Chemistry, Faculty of Chemistry and Biochemistry, 12853 Kanagawa University, 3-27-1 Rokkakubashi, Kanagawa-ku, Yokohama 221-8686, Japan; ‡ Institute for Integrated Radiation and Nuclear Science, Kyoto University, Osaka 590-0494, Japan; § Australian Centre for Neutron Scattering, Australian Nuclear Science and Technology Organization (ANSTO), Kirrawee DC, Sydney, NSW 2232, Australia; ∥ School of Chemistry, The University of Sydney, Sydney, NSW 2006, Australia; ⊥ Center for Basic Research on Materials, National Institute for Materials Science, Tsukuba 305-0044, Japan; # Department of Chemistry, Faculty of Science and Engineering, 12872 Kindai University, Higashi-Osaka, Osaka 577-8502, Japan; ∇ International Institute for Carbon Neutral Energy Research (WPI-I2CNER) and Department of Applied Chemistry, Faculty of Engineering, 12923 Kyushu University, 744 Motooka, Nishi-ku, Fukuoka 819-0395, Japan; ○ Department of Applied Chemistry, Graduate School of Engineering, Kyushu University, 744 Motooka, Nishi-ku, Fukuoka 819-0395, Japan

## Abstract

Oxy-hydroxides represent potential proton carriers for solid acid catalysts and proton conductors owing to their hydroxide-rich compositions. However, their applications in high-temperature environments are limited due to thermal instability associated with dehydration at moderate to high temperatures. Therefore, the development of oxy-hydroxides with enhanced thermal stability is of critical importance. Herein, we report the discovery of a strontium–gallium oxy-hydroxide, Sr_2_Ga_3_O_6_(OH), with exceptional thermal stability. The Sr–Ga oxy-hydroxide was successfully synthesized via an unconventional synthesis route, “*vapor hydroxidation*”, involving high-temperature heat treatment in highly concentrated water vapor. Structural characterization employing X-ray diffraction, neutron diffraction, and transmission electron microscopy revealed that the Sr–Ga oxy-hydroxide crystallizes in a trigonal structure (*R*3̅ space group) with lattice parameters *a* = 18.1904(2) Å and *c* = 7.2693(1) Å. Notably, OH^–^ anions are nonuniformly distributed within the crystal structure and are confined to a narrow space between two strontium sites. Thermogravimetry combined with desorption gas analysis indicated that OH^–^ anions are retained in the crystal structure up to approximately 850 °C. In situ infrared spectroscopy upon heating demonstrated proton redistribution via multilinked hydrogen bonds at elevated temperatures, which likely contributes to the excellent thermal stability.

## Graphical abstract

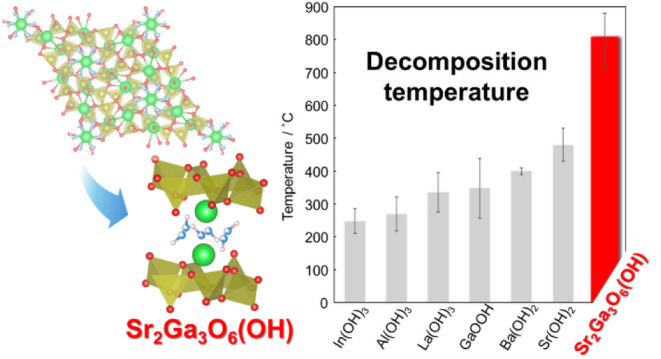

## Introduction

Inorganic solids, such as metal oxides, exhibit diverse functionalities that depend on their chemical composition and crystal structure. Research on various material functionalities, including dielectric^1^ and catalysis,^2,3^ has been actively conducted for their applications as practical ceramics. Metal hydroxides and oxy-hydroxides, which are inorganic compounds containing hydroxide (OH^–^) anions derived from water molecules, are also recognized as practical ceramics. Their chemical compositions, characterized by a high concentration of protons, make them promising candidates for proton-related functionalities.^4^ Despite their potential as proton carriers, these compounds present challenges for high-temperature applications, as dehydration induces phase decomposition into oxides at intermediate temperatures.^5–7^ For instance, while proton conductors exhibit higher ionic conductivity at lower temperatures compared to oxide-ion conductors, they require a humid atmosphere as a source of protons for operation. Moreover, their performance deteriorates significantly under high-temperature conditions due to proton loss.^8,9^ Similarly, in solid acid catalysts, proton loss at elevated temperatures diminishes Brønsted acidity, thereby reducing catalytic performance.^10^ Therefore, the development of (oxy-)­hydroxides with enhanced thermal stability is essential for advancing both fundamental and applied research in proton-related technologies.

(Oxy-)­hydroxides are generally synthesized via conventional liquid-phase methods, such as precipitation^11,12^ and hydrothermal methods.^13,14^ An autoclave-based synthesis approach employing a hydroxide hydrogel precursor has also been developed.^15^ These syntheses are conducted at low temperatures due to the inherent thermal instability of the metal–OH framework in these compounds. However, it is conceivable that unprecedented (oxy-)­hydroxides could be synthesized under unexplored reaction conditions involving concentrated water vapor at elevated temperatures. To enable this unique synthesis, which we term “*vapor hydroxidation*,” we have developed a reactor capable of performing high-temperature heat treatments under highly concentrated water vapor. This reactor generates a gas flow containing up to 100 vol % water vapor, approximately 40 times higher than the saturated vapor pressure at room temperature (2.4 vol %). Additionally, the system allows heat treatment above 400 °C, conditions that surpass the dehydration and decomposition temperatures of conventional (oxy-)­hydroxides and have therefore typically been avoided in previous studies.

Through the “*vapor hydroxidation*” method, which involves the heat treatment of a barium–indium oxide precursor under 80 vol % water vapor at 500 °C, we successfully synthesized a new oxy-hydroxide [Ba_2_O_*x*_(OH)_*y*_]_0.55_InO_2_ (referred to as “*mf*-BI”) that fundamentally differs from the widely known Ba–In oxy-hydroxide BaInO_2_(OH) with a perovskite-type structure.^16^ Structural analysis revealed that the resulting oxy-hydroxide crystallizes in a misfit-layered structure consisting of the incommensurate stacking of a barium hydroxide block and an indium oxide block. While the perovskite-type Ba–In oxy-hydroxide decomposes at approximately 300 °C, the newly synthesized oxy-hydroxide demonstrates exceptional thermal stability, retaining hydroxide ions up to 700 °C. In addition, this oxy-hydroxide functions as a proton conductor with an electrical conductivity of 5.0 × 10^–4^ S cm^–1^ at 500 °C even under dry argon.

Based on this finding, the combination of highly and slightly hydrophilic alkali-earth elements (such as barium) and aluminum-group elements (such as indium) may be promising for the exploration of new oxy-hydroxides using our high-temperature process, “*vapor hydroxidation*.” Moreover, the development and comparison of oxy-hydroxides with entirely different crystal structures provide valuable insights into the key factors governing their thermal properties, paving the way for further advancements in material design and various high-temperature applications.

In this study, a new oxy-hydroxide was identified in the combination of strontium and gallium, which are homologous elements for barium and indium, respectively. Nearly phase-pure samples were successfully synthesized by heat-treating Sr–Ga oxide precursors at 500 °C under approximately 80 vol % water vapor. Remarkably, the Sr–Ga oxy-hydroxide, Sr_2_Ga_3_O_6_(OH), exhibits exceptionally high thermal stability, with a robust hydroxide framework remaining nearly intact up to 850 °C. The crystal structure and O–H bonding nature of Sr_2_Ga_3_O_6_(OH) were thoroughly investigated, and the origin of its exceptional thermal stability was discussed.

## Experimental Section

### Synthesis

Samples of Sr–Ga oxide precursors were synthesized via the citrate-lactate route. Sr­(NO_3_)_2_ (99.5%, Kanto Chemical) and Ga­(NO_3_)_3_·*n*H_2_O (99.999%, Kojundo Chemical Laboratory; the *n* value was determined to be 6.82 by thermogravimetry; Figure S1 of the Supporting Information) were used as starting materials. These reagents were weighed to achieve a composition ratio of Sr/Ga = 0.67, dissolved in an aqueous solution containing citric acid in a 3-fold molar excess, and heated at 120 °C to promote gelation. The resulting gel product was calcined in air at 450 °C for 1 h and 600 °C for 1 h, followed by firing at 1000 °C for 10 h and subsequently at 1200 °C for 10 h to obtain the oxide precursor, based on an established protocol. The resulting precursor was pressed into pellets under a uniaxial pressure of 5 MPa and heat-treated in approximately 80 vol % water vapor at 500 °C for 3 h using a custom-built reactor, as schematically depicted in Figure S2 of the Supporting Information (SI). The highly concentrated water vapor was generated by directly injecting 64 μL min^–1^ of liquid water—corresponding to approximately 80 mL min^–1^ of water vapor at room temperature—into a vertical silica reactor with a flowing N_2_ gas rate of 20 mL min^–1^. The resulting water-vapor-treated sample was postannealed in flowing N_2_ gas at 600 °C for 1 h. Deuterium-containing samples were synthesized similarly using D_2_O (99.8%, ISOTEC).

### Characterizations

The phase purity of the products was analyzed using X-ray powder diffraction (XRD, Ultima IV Protectus; Cu Kα radiation, Rigaku). Measurements were performed over a 2θ range of 5–130° with a step width of 0.02° min^–1^ and a scanning speed of 5° min^–1^. For each diffraction pattern, constituent phases were identified based on the International Centre for Diffraction Data (ICDD) PDF and the Inorganic Crystal Structure Database (ICSD). Rietveld refinement was performed using the RIETAN-FP software^17^ based on the space group and atomic coordinates of the model compound.

Powder neutron diffraction (ND) experiments were conducted using high-resolution diffractometer ECHIDNA at ANSTO (Australia) to refine the crystal structure with precise O^2–^ and OH^–^ configurations. A powder sample was loaded into a 6 mmϕ vanadium cell with a height of 18 mm. Data acquisition was performed at 3 K over a scanning range of 5 to 155°. Rietveld refinement of the ND data was performed using the FullProf Suite,^18^ and the resulting crystal structure was visualized with the VESTA software.^19^

Crystallographic features and atomic arrangements of the target compound were investigated using scanning transmission electron microscopy (STEM) with a Thermo Fisher Scientific Titan Cubed microscope. A portion of the powder product was ground in a glovebox filled with dry N_2_ gas (dew point approximately −10 °C), dispersed in methanol by ultrasonication, and then placed on a microgrid. Observations were conducted under the following conditions: for TEM imaging and electron diffraction (ED) analysis, an acceleration voltage of 300 kV and a camera length of 285 nm; for STEM imaging, an acceleration voltage of 300 kV, a STEM camera length of 115 nm, a convergence semiangle of 17.9 mrad, a BF semiangle of 4.4 mrad, an ABF semiangle of 8.2–17 mrad, and a HAADF semiangle of 46 −200 mrad. The observed ED patterns were compared with those simulated using CrystalMaker (CrystalMaker Software Ltd.) and SingleCrystal (CrystalMaker Software Ltd.).

To investigate phase changes in the target compound as a function of temperature, high-temperature synchrotron radiation X-ray diffraction (HT-SXRD) measurements were conducted at BL13XU of SPring-8 (Japan). A powder sample was placed in a sapphire cell^20^ containing a hole. The sample was heated with a rate of 10 K min^–1^, and the N_2_ flow rate was maintained at 24 mL min^–1^. The measurements were performed using a two-dimensional CdTe detector (Lambda 750 K) with an X-ray wavelength (λ) of 0.49592 Å over a scanning range of 2θ = 1° to 40°, while the sample was heated from room temperature to 1000 °C in 10 °C increments under flowing N_2_ gas.

Thermogravimetric (TG) analysis (Thermo Plus TG-8122, Rigaku) was employed to investigate the thermal behavior of the target compound. The sample was loaded into an open-type Al_2_O_3_ sample pan. Measurements were conducted from room temperature to 1000 °C under an N_2_ atmosphere. Desorbed gas species at elevated temperatures were analyzed using a quadrupole mass spectrometer (Q-MS; Transpector CPM, INFICON) under a flowing N_2_ gas (10 mL min^–1^) with a heating rate of 10 °C min^–1^.

A Fourier transform infrared spectrometer (FT/IR-4700, JASCO) equipped with a DALTGS detector and a CaF_2_ window plate, along with a heated diffuse reflector (DR-650Ai, JASCO), was employed to investigate the chemical nature of O–H bonds in the target compound. The sample was loaded into a SUS holder and mounted on the sample stage. IR spectra were collected over a wavenumber range of 1000 to 4000 cm^–1^ under a flowing N_2_ gas (150 mL min^–1^) at temperatures ranging from 40 to 950 °C. A heating rate of 20 °C min^–1^ was applied, with a holding time of 3 hours at each temperature. Baseline correction was performed using Spectrum Manager (JASCO) to eliminate contributions from residual H_2_O and CO_2_ gases. Peak deconvolution of the O–H stretching spectral oscillations was carried out using OriginPro (OriginLab) with a Voigt function, which represents the convolution of Gaussian and Lorentz distributions. The parameters obtained from this analysis are provided in the Supporting Information (SI).

## Results and Discussion

### Crystal Structure

As presented in Figure 1, the resulting Sr–Ga (Sr/Ga = 0.67) oxide precursor primarily contains a triclinic Sr_3_Ga_4_O_9_ phase.^21^ The reaction of this oxide precursor under highly concentrated water vapor at 500 °C leads to diffraction patterns that cannot be attributed to known Sr–Ga oxides or (oxy-)­hydroxides, suggesting the formation of a new compound, possibly an oxy-hydroxide. To optimize the synthesis conditions for this compound, heat treatments of the oxide precursor were conducted at 300, 500, and 700 °C for 3 h under water vapor concentrations of 30, 50, 80, and 100 vol %. As demonstrated in Figure S3 of the SI, the target compound preferentially forms in samples synthesized under 80–100 vol % water vapor. In contrast, the characteristic diffraction pattern disappears when the material is heat-treated at 300 or 700 °C in 30–50 vol % water vapor. Based on these results, the optimal synthesis condition is presumed to be at 500 °C in 80 vol % water vapor. Despite optimization, trace amounts of Sr_3_Ga_2_(OH)_12_ and SrCO_3_ are observed as secondary phases. However, postannealing under flowing N_2_ gas at 600 °C effectively eliminates the Sr_3_Ga_2_(OH)_12_ impurity while preserving the primary phase. Furthermore, no phase decomposition or degradation of crystallinity was observed, even after N_2_ annealing at 600 °C for 1 week (Figure S4). The synthesis of the Sr–Ga oxy-hydroxide with a Sr/Ga ratio of 0.67 was attempted using conventional hydrothermal and solvothermal methods, both of which are widely employed techniques. However, these approaches failed to yield the target phase, as demonstrated in the SI (Table S1 and Figure S5). This result suggests that the formation of the oxy-hydroxide is uniquely enabled by the “*vapor hydroxidation*” process.

**Figure 1:**
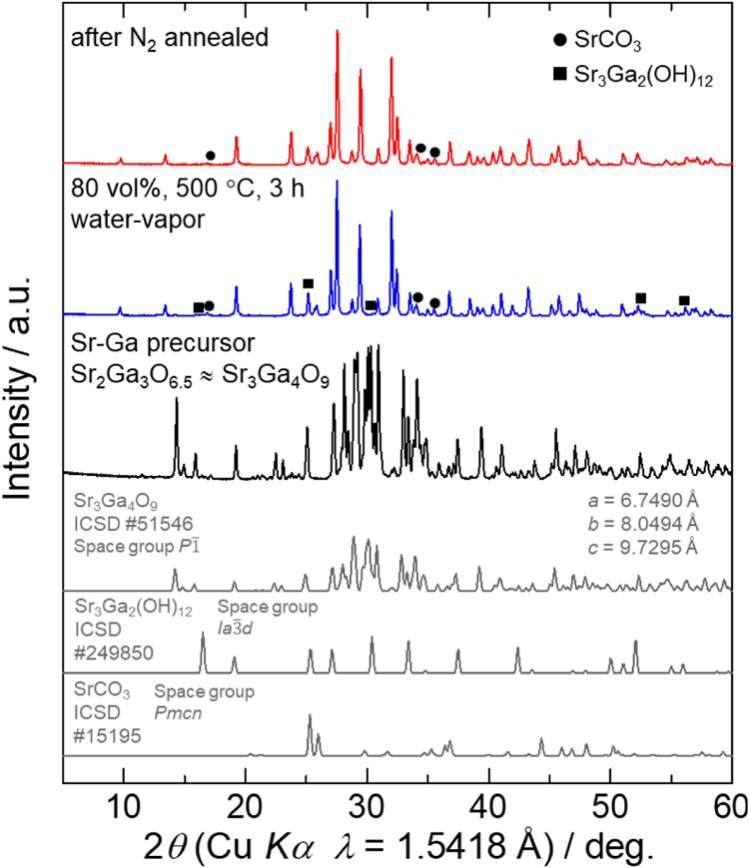
XRD patterns for the Sr–Ga (Sr/Ga = 0.67) oxide precursor (black), as-synthesized sample in a concentrated water-vapor atmosphere (blue), and N_2_-annealed sample (red). Simulated patterns for Sr_3_Ga_4_O_9_ (ICSD #51546), Sr_3_Ga_2_(OH)_12_ (ICSD #249850), and SrCO_3_ (ICSD #15195) are also presented.

A model compound for crystal structure determination was identified within the ICSD database. As described later, the TG analysis of the target compound during heating under flowing N_2_ gas reveals approximately 2.2 wt % of water loss between 600 and 800 °C, leading to its decomposition into an oxide with a nominal composition of Sr_2_Ga_3_O_6.5_. Assuming that the entire weight loss arises from water loss, the O^2–^/OH^–^ anionic ratio is estimated to be Sr_2_Ga_3_O_5.89_(OH)_1.22_, suggesting that the target compound may be an oxy-hydroxide with an ideal composition expressed as Sr_2_Ga_3_O_6_(OH). The ICSD database (Data Release 2024.2) contains 13 compounds with the compositional ratio “A_2_B_3_X_1_Y_6_”, and notably, the diffraction pattern for Sr_2_Al_3_O_6_F (ref 22; ICSD #36690) closely resembles that of the target compound. This oxy-fluoride crystallizes in a trigonal structure with the *R*3̅ space group (#148), and its Eu^2+^-for-Sr^2+^ substituted derivatives have been previously investigated for their luminescence properties.^23^ Given the compositional similarity, this oxy-fluoride may serve as a potential model compound for structural refinements of our oxy-hydroxide. It is worth noting that OH^–^ and F^–^ anions share similarities in both oxidation state and ionic radius (1.37 Å and 1.33 Å, respectively, for CN: VI),^24^ implying that OH^–^ can be considered a “pseudo-halide” analog of F^–^.

Figure 2 and Table S2 summarize the Rietveld analysis refinement result obtained from room temperature XRD data. The Sr_2_Ga_3_O_6_(OH) pattern was analyzed by applying the Sr_2_Al_3_O_6_F model, and a two-phase refinement was performed to account for the presence of SrCO_3_, resulting in good agreement between the experimental and calculated patterns. Their weight percentages in the sample are determined to be 94.6 and 5.4 wt %, respectively. The refined lattice parameters for Sr_2_Ga_3_O_6_(OH) are *a* = 18.226(1) Å and *c* = 7.2830(3) Å in the hexagonal setting. The structural model includes two crystallographically inequivalent sites for both strontium and gallium, while five anionic sites are present in the crystal structure (Figure S6). Among these, O^2–^ and OH^–^ anions are indistinguishable due to the invisibility of hydrogen atoms in XRD analysis.

**Figure 2:**
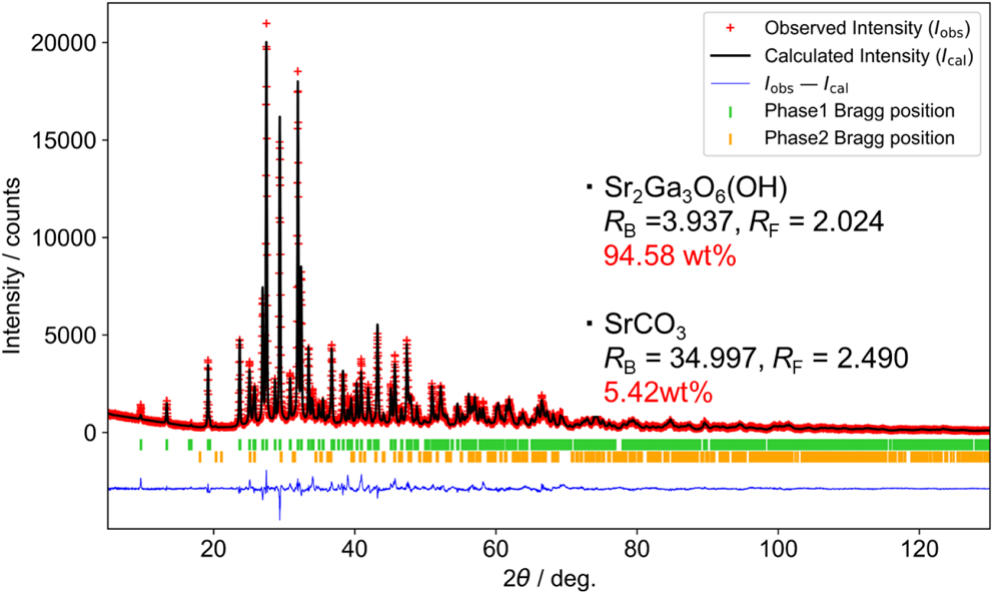
Rietveld analysis result using an XRD pattern.

Next, ND analysis was employed to determine the O^2–^ and OH^–^ arrangements. Measurements were conducted on Sr_2_Ga_3_O_6_(OH) with light hydrogen (referred to as “H sample”; Figure S7­(a)) at 3 K to minimize the atomic displacement due to thermal vibrations. A deuterium-substituted sample, Sr_2_Ga_3_O_6_(OD) (referred to as “D sample”; Figure S7­(b)), was also examined; however, the H sample yielded more accurate results than the D sample. This is attributed to the negative neutron scattering length of H, which provided higher contrast with the rest of the structure (Sr, Ga, and O have positive neutron scattering lengths) and reduced interference with other parameters. The refinement was performed with sequential refinement of isotropic atomic displacement parameters (*B*), atomic positions, occupancies, and anisotropic atomic displacement parameters (*U*). As shown in Figure 3, the ND data are well reproduced when hydrogen atoms are positioned near the Sr2 site. Atomic parameters and refined details are summarized in Table S3. The refinement yields *R*_p_ = 2.05% and *R*_wp_ = 2.51%, while the goodness-of-fit χ^2^ (GOF) is 3.44. The resulting Bond Valence Sum (BVS)^25^ values are 1.84 (Sr1), 2.51 (Sr2), 3.12 (Ga1), and 2.99 (Ga2), with the Sr2 site exhibiting a relatively high effective charge.

**Figure 3:**
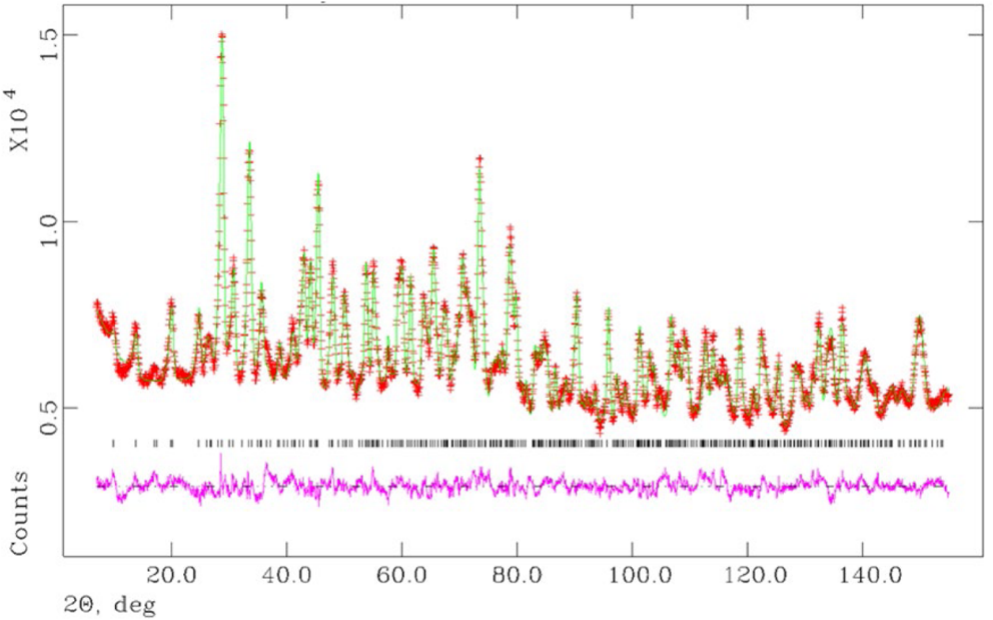
Rietveld refinement result for the ND data of Sr_2_Ga_3_O_6_(OH) at 3 K.

The refined crystal structure of Sr_2_Ga_3_O_6_(OH), is schematically illustrated in Figure 4. This oxy-hydroxide crystallizes in a structure essentially identical to that of Sr_2_Al_3_O_6_F, with Ga^3+^ substituting Al^3+^ and OH^–^ replacing F^–^. Hydrogen atoms bind to oxygen at the O5 site, forming OH^–^ anions in the Sr_2_Ga_3_O_6_(OH) structure. This O5 site corresponds to the fluorine site in the isostructural Sr_2_Al_3_O_6_F. Consequently, OH^–^ anions are nonuniformly distributed in the crystal structure, confined within a narrow space between two Sr2 sites. We consider that the elevated BVS value observed at the Sr2 site, relative to the formal valence, is likely attributable to the reduced negative charge on the oxygen atom in hydroxide anions, which results in a shorter Sr2–O bond distance. Notably, a similarly enhanced BVS at the Sr2 site has also been reported in the isostructural Sr_2_Al_3_O_6_F, where the high electronegativity of fluorine was proposed as a contributing factor to the increased BVS at the Sr2 site.^22^

**Figure 4:**
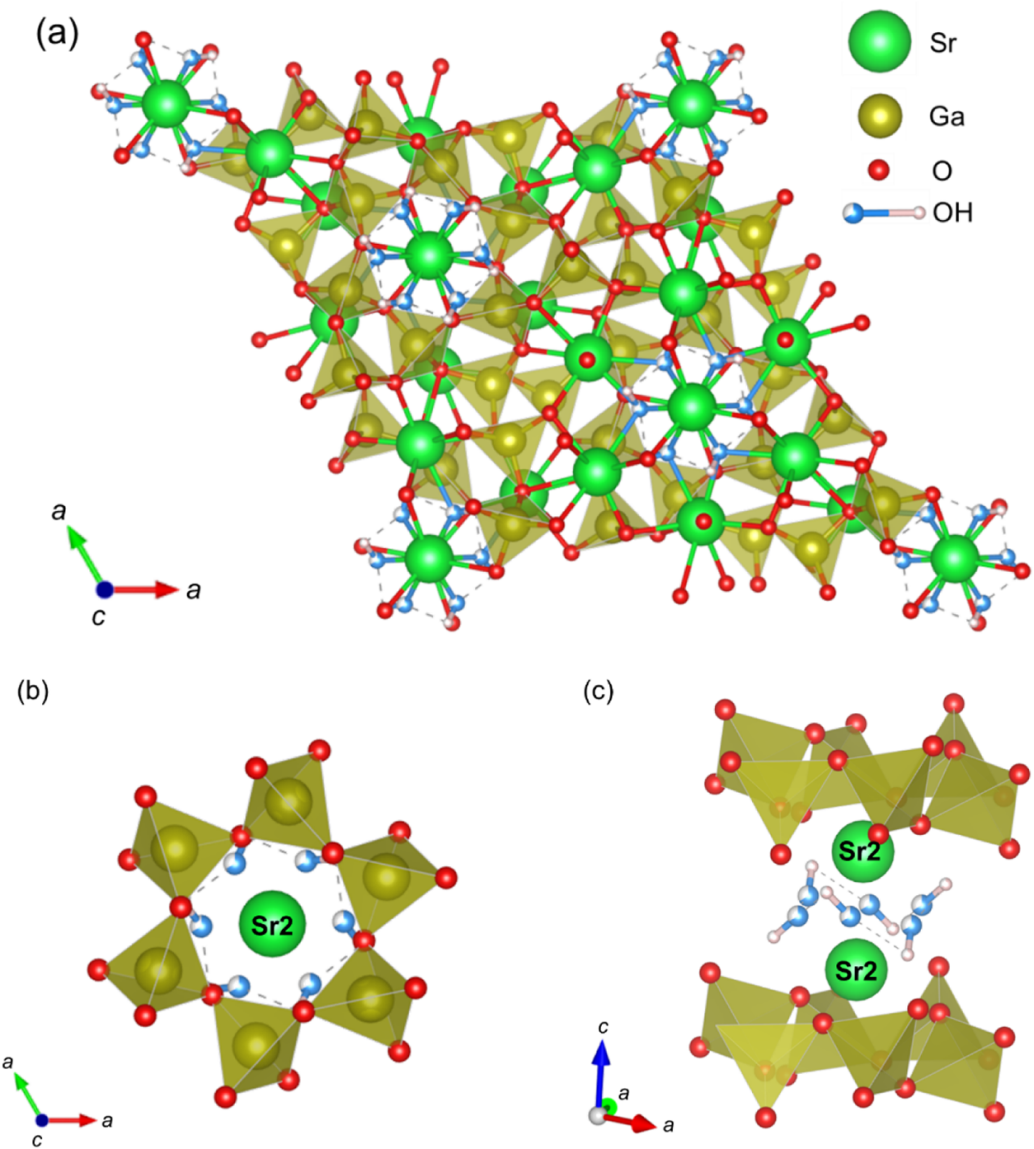
Crystal structure of Sr_2_Ga_3_O_6_(OH) with refined atomic positions. (a) Crystal structure viewed along the *c*-axis. (b, c) Highlighted atomic arrangements of OH^–^ anions. These illustrations were drawn using the VESTA software.^19^

The STEM-ABF/ADF images in Figure 5 verify the hexagonal arrangement of Sr and Ga cations, showing excellent agreement with the refined structure along the crystallographic *c*-axis. It should be noted that ADF images display atomic columns as bright spots, with intensities approximately proportional to the atomic number *Z* to the power of 1.5 to 1.7,^26^ allowing straightforward assignment of the Sr and Ga atomic positions. The TEM image reveals rhombohedral single-crystal grains, and the selected-area electron diffraction (SAED) pattern is consistent with the simulated pattern generated from our ND structural model (Figure S8). The atomic arrangement of Sr and Ga is discernible in the ABF image, whereas light elements such as O and H remain undetectable due to the low probe current used during measurement. Attempts to enhance probe currents were unsuccessful, as the oxy-hydroxide exhibited chemical instability under intense electron irradiation.

**Figure 5:**
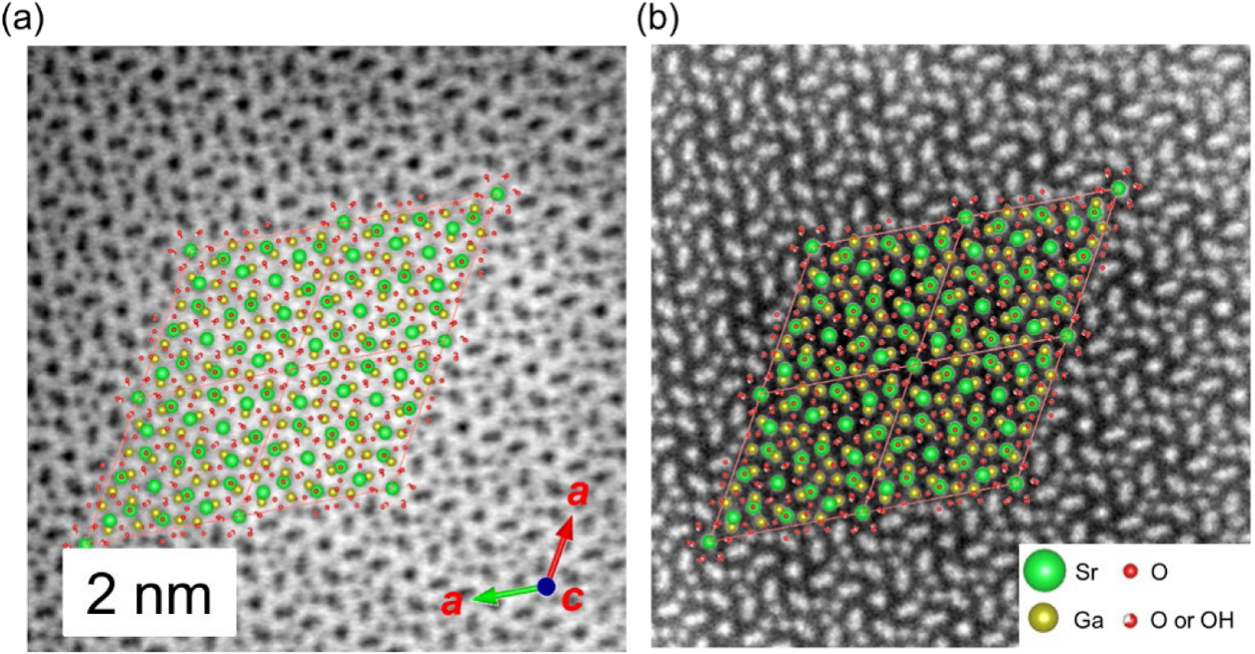
High-resolution STEM images of Sr_2_Ga_3_O_6_(OH). (a) ABF image and (b) ADF image.

It is noteworthy that Sr_2_Ga_3_O_6_(OH) possesses a crystal structure characterized by a spatially heterogeneous proton distribution. This unique structure likely arises from the combination of two metallic elements with distinct chemical characteristics: the highly hydrophilic strontium preferentially binds to hydroxide anions, while less hydrophilic gallium forms an oxide network. The spatial distribution of protons (or hydroxide anions) within a compound is expected to intimately influence its proton transport kinetics. Indeed, an AC-impedance measurement of Sr_2_Ga_3_O_6_(OH) reveals poor electrical conductivity even at elevated temperatures (Figures S9­(a–c) and Table S4), in good agreement with its crystallographic nature, which is characterized by strong proton confinement near the Sr2 site. Notably, [Ba_2_O_*x*_(OH)_*y*_]_0.55_InO_2_ (*mf*-BI), another oxy-hydroxide obtained via our “*vapor hydroxidation*” method, also exhibits heterogeneous proton distribution in the crystal structure.^16^ However, its alternate stacking of barium hydroxide and indium oxide blocks facilitates the formation of effective proton diffusion pathways, resulting in moderate proton conductivity.

### Thermal Behaviors and Local Environment around Hydroxide Anions

TG combined with MS analysis (Figure 6) reveals that Sr_2_Ga_3_O_6_(OH) undergoes two distinct weight loss steps: from 100 to 300 °C and from 600 to 900 °C. These weight losses are primarily attributed to water desorption (*m*/*z* = 18). The minor weight loss between 100 and 300 °C corresponds to the desorption of water molecules adsorbed on the sample surface, while the significant weight loss between 600 and 900 °C is attributed to the collapse of the oxy-hydroxide framework into an oxide. Additionally, at approximately 800 °C, a CO_2_ (*m*/*z* = 44) signal is observed, likely due to the decomposition of residual SrCO_3_ impurities. HT-SXRD measurements demonstrate that the crystal structure of Sr_2_Ga_3_O_6_(OH) remains stable up to 800 °C, while structural collapse is observed above 900 °C (Figure S10­(a)), highlighting its excellent thermal stability.

**Figure 6:**
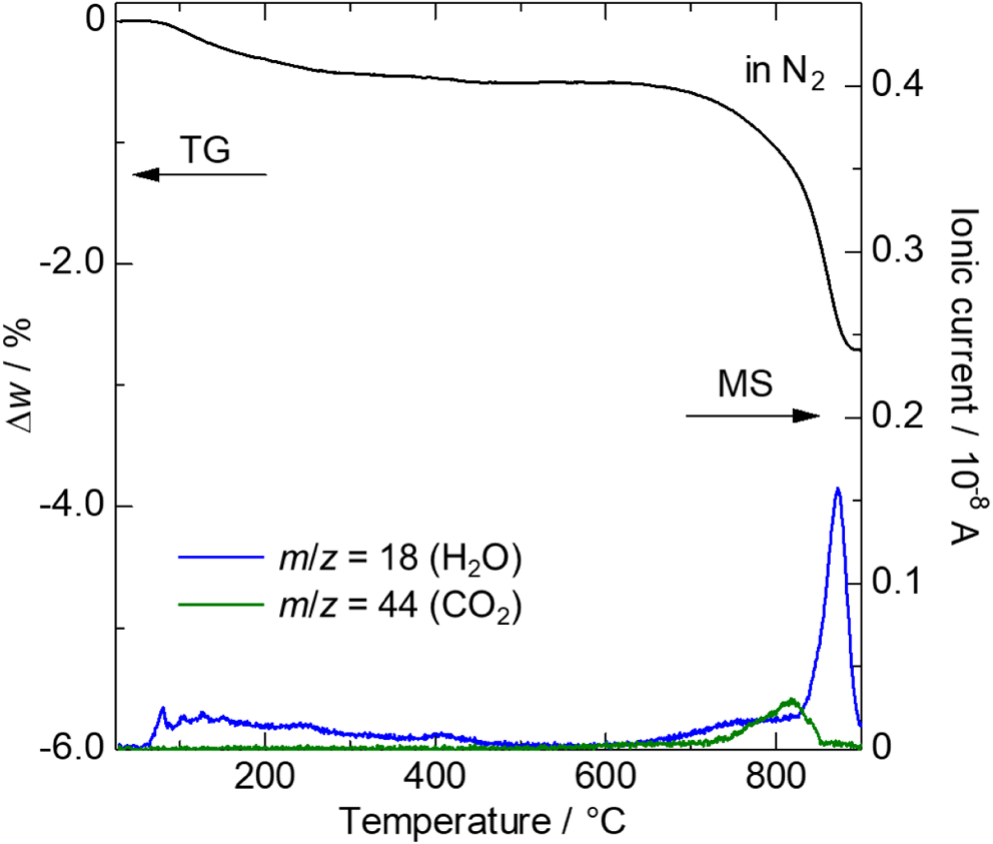
Results of TG and Q-MS analyses of Sr_2_Ga_3_O_6_(OH). The data were measured upon heating in flowing N_2_ gas.

The decomposition temperature of Sr_2_Ga_3_O_6_(OH) is significantly higher than those of simple (oxy-)­hydroxides; see the summarized TG data in Figure S11, and far exceeds that of perovskite-type oxy-hydroxides, such as Ba­(Zr, Y)­O_3−δ−*y*_(OH)_2*y*_ ^6^ and layered double hydroxides (LDHs).^27,28^ Calcium phosphate apatite (CPAp) is another compound known to retain OH^–^ at high temperatures, with dehydration occurring between 100 and 1200 °C.^29^ During dehydration, CPAp maintains its apatite structure while the sample weight decreases linearly, indicating the gradual release of OH^–^. In contrast, for Sr_2_Ga_3_O_6_(OH), the sample weight, i.e., the OH^–^ content, remains nearly constant up to 600 °C. A key difference between Sr_2_Ga_3_O_6_(OH) and CPAp may lie in the position of OH^–^ anions in the structure; Sr_2_Ga_3_O_6_(OH) consists of a robust oxy-hydroxide framework that suppresses OH^–^ release, whereas CPAp dehydrates more easily, as OH^–^ anions are incorporated into atomic channels within the apatite-type phosphate framework.

A more detailed HT-SXRD analysis (Figure S10­(b,c)) shows that the 012 and 202 diffraction peaks shift to lower angles more prominently at elevated temperatures. The *c*-axis length indeed exhibits a larger increment above 600 °C. This elongation is also observed in the sample postannealed at 600 °C for 1 week, as shown in Figure S4. Given the coincidence in the onset temperatures of lattice expansion and weight loss, this behavior may be correlated with a slight structural change associated with the partial release of OH^–^ anions.

### O–H Bonding Nature in the Crystal Structure

The excellent thermal stability of Sr_2_Ga_3_O_6_(OH) warrants attention, and a detailed investigation of the O–H bonding nature may provide insight into its origin. In the FT-IR spectrum of the H sample, a broad absorption peak attributed to the O–H stretching mode^30–33^ is observed between approximately 2700 and 3700 cm^–1^ (Figure 7). Similarly, in the D sample, a peak corresponding to the O–D stretching mode appears between 2200 and 2700 cm^–1^, consistent with the H/D isotope effect (Figures S12 and S13).^34,35^ The broad peak of the O–H stretching mode consists of both sharp and broad components, indicating the presence of multiple forms of O–H bonds within the crystal structure. Upon heating, the peak intensity exhibits complex evolution, with both increasing and decreasing intensities, finally converging into a broad peak at 3400 cm^–1^ at elevated temperatures. These results suggest that the nature of O–H bonding is strongly temperature-dependent. Above 800 °C, the overall peak intensity decreases significantly, in good agreement with the TG data presented in Figure 6.

**Figure 7:**
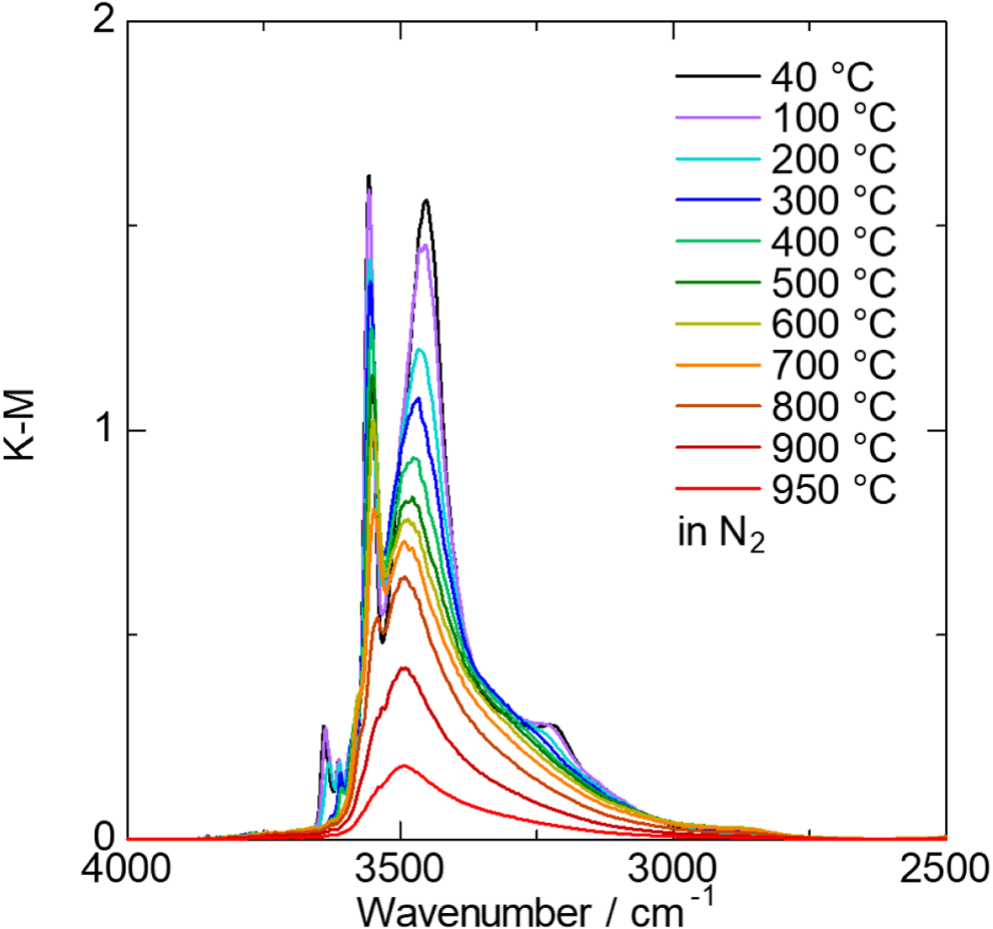
In situ FT-IR spectra of Sr_2_Ga_3_O_6_(OH) upon heating from 40 to 950 °C in flowing N_2_ gas.

The main component of the O–H stretching mode spectrum at 40 °C is reasonably reproduced using five peaks of a Gaussian–Lorentzian mixed function labeled Peak #1 to #5, as shown in Figure 8. The position and half-width of each component obtained from spectral fitting are summarized in Table S5 (the result of the D sample in Table S6). For the assignment of each spectral component in Sr_2_Ga_3_O_6_(OH), previously reported data on CPAp^36–38^ are informative and serve as a reference. The crystal structure of CPAp consists of tetrahedral PO_4_^3–^ units and triangular Ca^2+^ units aligned along the *c*-axis, forming a Ca-channel. An OH^–^ anion within the Ca-channel forms a strong O–H bond, which corresponds to a narrow component at 3572 cm^–1^.^39^ Hydrogen bonds formed between hydrogen of OH^–^ anions and oxygen in neighboring PO_4_^3–^ tetrahedra are elongated^40–44^ and, therefore, appear as a broad peak component at lower wavenumbers, ranging from 2400 to 3400 cm^–1^.^39^ Based on these findings, the narrow component observed at higher wavenumbers in Sr_2_Ga_3_O_6_(OH), Peak #1, is attributed to O–H stretching within an isolated OH^–^ anion, while Peaks #2, #4, and #5, which appear at lower wavenumbers and exhibit broader profiles, are assigned to hydrogen bonds. Notably, the correlation between peak position and hydrogen bond length reported in the literature^45^ yields estimated O···H distances of 2.1, 1.9, and 1.8 Å for Peaks #2, #4, and #5, respectively, in agreement with the atomic arrangement of Sr_2_Ga_3_O_6_(OH) as discussed later in our structural model. The assignment of Peak #3 remains uncertain; however, considering its temperature-dependent spectral weight similar to that of Peak #1, we attribute this component to O–H stretching within a single OH^–^ anion that is slightly perturbed by the presence of neighboring OH^–^ anions.

**Figure 8:**
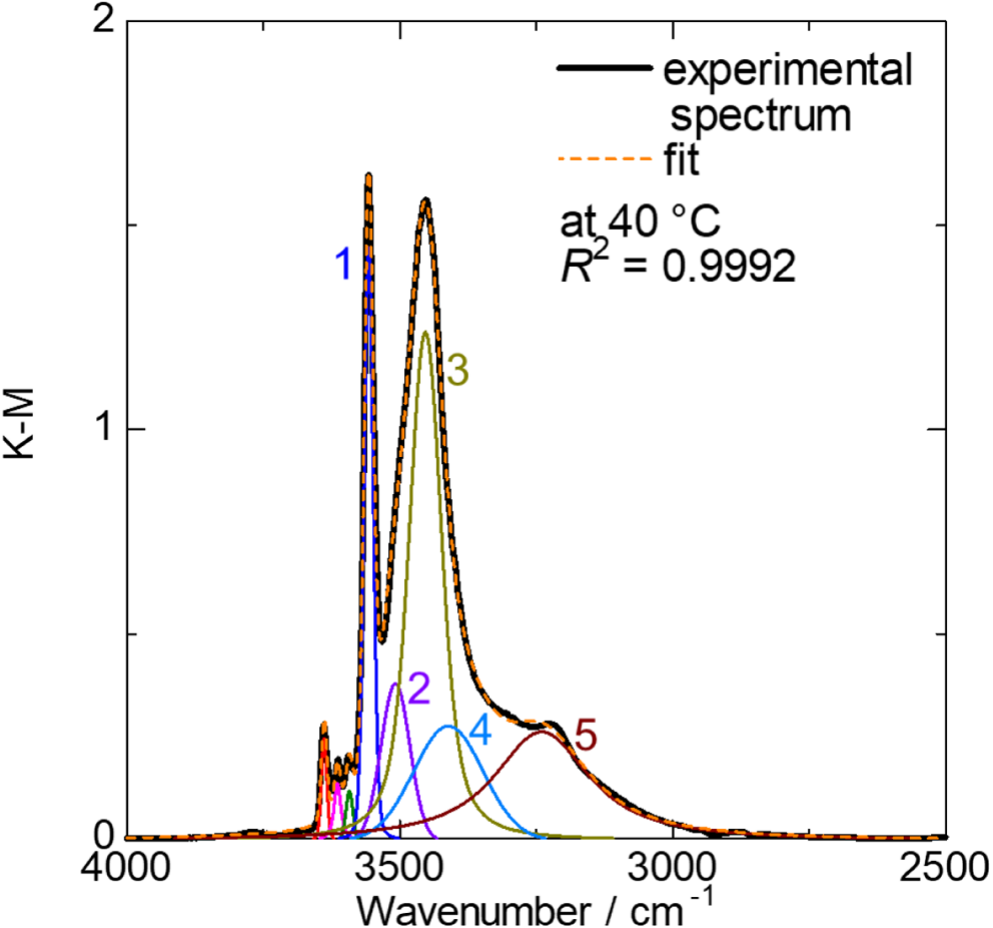
Deconvoluted in situ FT-IR spectra of Sr_2_Ga_3_O_6_(OH) at 40 °C.

Let us discuss the local atomic configuration surrounding OH^–^ anions in the Sr_2_Ga_3_O_6_(OH) crystal structure. As already mentioned and schematically illustrated in Figures 4(b,c), OH^–^ anions are clustered and confined within a narrow space between two Sr2 sites. Unlike fluoride anions in the isostructural Sr_2_Al_3_O_6_F, OH^–^ anions in Sr_2_Ga_3_O_6_(OH) form strong hydrogen bonds. Based on the ND data, the interatomic distance within the OH^–^ anion is 1.02(3) Å, while the hydrogen bond lengths are 1.88(3) Å between two neighboring OH^–^ anions and 2.05(2) Å between OH^–^ and the nearest GaO_4_ tetrahedron. These values are within the typical range observed in inorganic compounds with hydrogen bonds.^46,47^ The OH^–^ site in Sr_2_Ga_3_O_6_(OH) is not fully occupied, with an occupancy of *g* = 4/6 due to a compositional constraint, and the exact local OH^–^ arrangement remains unknown. To quantitatively assess the chemical stability of this compound, the chemical bonding energy must be precisely calculated for each atomic configuration.

Temperature-dependent variations in the peak areas of each component are presented in Figure 9. The intra–OH stretching components, assigned as Peaks #1 and #3, decrease rapidly with increasing temperature, similar to observations reported for hydroxide-incorporated BaTiO_3_ synthesized via hydrothermal methods.^48^ In contrast, the hydrogen bond components, Peaks #2, #4, and #5, exhibit distinct temperature dependencies. This complex behavior arises from a redistribution of spectral weight among multiple components, potentially indicating the existence of itinerant protons. Notably, an increase in spectral weight with increasing temperature is also observed in the thermally stable oxy-hydroxide Ba­(Zn_*x*_Nb_1–*x*_)­O_3−δ−*y*_(OH)_2*y*_,^33^ suggesting that this feature may be a signature of enhanced hydrogen bonding within the structural framework.

**Figure 9:**
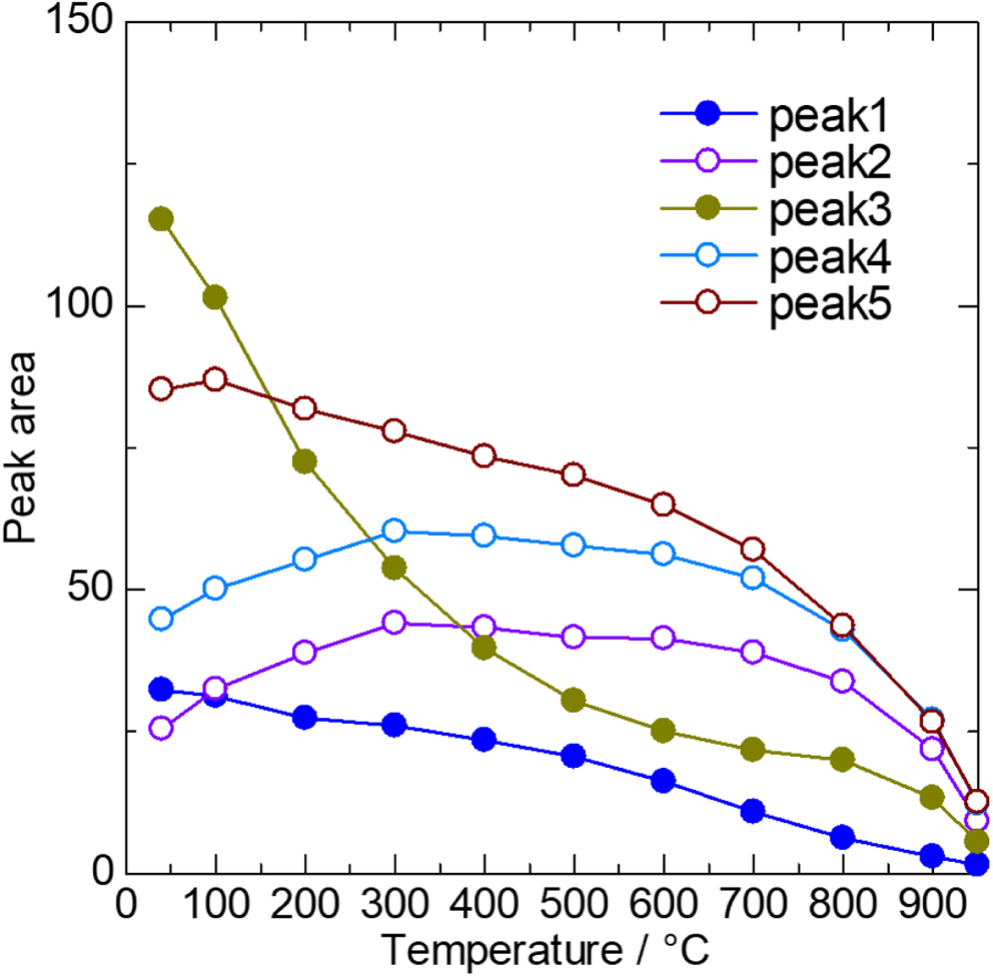
In situ FT-IR peak areas as a temperature change of Sr_2_Ga_3_O_6_(OH).

The uniqueness of Sr_2_Ga_3_O_6_(OH) is highlighted when compared with Sr_3_Ga_2_(OH)_12_, a potential reference compound composed of the same elements. As demonstrated in Figure S14­(a), the O–H stretching spectra of Sr_3_Ga_2_(OH)_12_ differ significantly from those of Sr_2_Ga_3_O_6_(OH). Unlike Sr_2_Ga_3_O_6_(OH), the spectra of Sr_3_Ga_2_(OH)_12_ display a broad peak in the 2500 – 3700 cm^–1^ range, and the peak intensity decreases rapidly with increasing temperature from 40 to 600 °C, while retaining its overall spectral profile (Figure S14­(b)). The persistence of similar spectral shapes across a wide temperature range indicates that the proton distribution remains unchanged with temperature. Based on the spectral profile and peak position, it is suggested that the protons in this compound are loosely bound via several hydrogen bonds^49,50^ and are subject to dynamic motion within the structural framework. This interpretation may be supported by the crystallographic features of Sr_3_Ga_2_(OH)_12_ (Figure S15),^51^ which reveal the absence of specific proton positions with distinct stabilities, resulting in a facile dehydration reaction at relatively low temperatures.

In contrast, proton redistribution occurs for Sr_2_Ga_3_O_6_(OH) during the heating process. Given that the spectral weight at lower frequencies associated with the O–H stretching mode becomes predominant at elevated temperatures, hydrogen bonding is likely a primary contributor to the exceptional thermal stability of this compound. We propose that the confined proton near the Sr2 sites in Sr_2_Ga_3_O_6_(OH) forms multilinked hydrogen bonds with a surrounding narrow cage composed of oxygen atoms from OH^–^ anions and/or the Sr–Ga–O framework, resulting in stronger chemical interactions than those involving dynamically mobile protons.

The findings of this study offer an effective strategy for designing thermally stable oxy-hydroxides: the confinement of OH^–^ anions (protons) within a narrow oxide framework, maximizing hydrogen bonding interactions to suppress dehydration and prevent structural collapse. The resulting oxy-hydroxides warrant attention as materials with new functionalities. The TG-MS data (Figure 6), combined with the HT-SXRD results (Figure S10), reveal that protons (OH^–^ anions) in the structural framework become thermally activated at temperatures above 700 °C. Conventional solid acid catalysts are typically used below their proton desorption temperatures, ranging from approximately 250 to 460 °C.^52,53^ Given that Sr_2_Ga_3_O_6_(OH) is anticipated to function as a proton donor or acceptor at elevated temperatures, this compound may serve as a new solid Brønsted acid catalyst for high-temperature reactions.

## Conclusions

A strontium–gallium oxy-hydroxide, Sr_2_Ga_3_O_6_(OH), was successfully synthesized employing our synthesis technique “*vapor hydroxidation*”, which involves high-temperature heat treatment in highly concentrated water vapor. This oxy-hydroxide crystallizes in a structure isotypic to the trigonal Sr–Al oxy-fluoride Sr_2_Al_3_O_6_F, with Ga^3+^ substituting Al^3+^ and OH^–^ replacing F^–^. Detailed structural refinements based on neutron diffraction analysis revealed that OH^–^ anions are nonuniformly distributed in the crystal structure, confined within a narrow space between two strontium sites. Thermogravimetry combined with desorbed gas analysis indicated that Sr_2_Ga_3_O_6_(OH) exhibits significant thermal stability, retaining OH^–^ anions in its crystal structure up to approximately 850 °C. Temperature-dependent infrared spectroscopy demonstrated proton redistribution via multilinked hydrogen bonds at elevated temperatures, likely contributing to the excellent thermal stability. Owing to these characteristics, Sr_2_Ga_3_O_6_(OH) holds promise as a novel solid Brønsted acid catalyst capable of proton donation and acceptance in high temperature environments—an ability that is difficult to achieve with conventional solid acid catalysts.

## Supplementary Material



## Notes

Synthesis procedure details; optimization of the reaction conditions for “*vapor hydroxidation*”; XRD patterns for samples postannealed for an extended duration; results of sample synthesis via hydrothermal/solvothermal methods; XRD and ND data analyses; results of the TEM observation, electrical conductivity measurement, and high-temperature synchrotron XRD study; thermal behaviors of (oxy-)­hydroxides studied by thermogravimetry; in situ FT-IR spectra of the deuterium-substituted sample and Sr_3_Ga_2_(OH)_12_, a reference compound (PDF)
