# Supplementary material for: A Sr–Ga Oxy-Hydroxide with High Thermal Stability: Unraveling Its Characteristic Hydrogen-Bond Network
Source: Inorg Chem. 2025 Aug 31;64(36):18294–303. doi: 10.1021/acs.inorgchem.5c02586 (PMC12442062; doi:10.1021/acs.inorgchem.5c02586)
Supplement: Supplementary file 1 [file ic5c02586_si_001.pdf]

Supporting Information

A Sr-Ga Oxy-Hydroxide with High Thermal Stability: Unraveling Its Characteristic Hydrogen-Bond Network

Yusuke Asai [a], Yuto Nishihara [a], Yoko Kokubo [a], Kenji Arai [a], Kosaku Ohishi [a], Satoshi Ogawa [a], Miwa Saito [a], Yusuke Nambu [b], Maxim Avdeev [c], Koji Kimoto [d], Zi Lang Goo [e], Kunihisa Sugimoto [e], Miki Inada [f, g], Katsuro Hayashi [g], Teruki Motohashi [a] *

[a] Department of Applied Chemistry, Faculty of Chemistry and Biochemistry, Kanagawa University, 3-27-1 Rokkakubashi, Kanagawa-ku, Yokohama, 221-8686, Japan

[b] Institute for Integrated Radiation and Nuclear Science, Kyoto University, Osaka 590-0494, Japan

c Australian Centre for Neutron Scattering, Australian Nuclear Science and Technology Organization (ANSTO), Kirrawee DC, NSW 2232, Australia; School of Chemistry, The University of Sydney, Sydney, NSW 2006, Australia

[d] Center for Basic Research on Materials, National Institute for Materials Science, Tsukuba 305-0044, Japan

[e] Department of Chemistry, Faculty of Science and Engineering, Kindai University, Higashi-Osaka, Osaka 577-8502, Japan

[f] International Institute for Carbon Neutral Energy Research (WPI-I2CNER) and Department of Applied Chemistry, Faculty of Engineering, Kyushu University, 744 Motooka, Nishi-ku, Fukuoka 819-0395, Japan

[g] Department of Applied Chemistry, Graduate School of Engineering, Kyushu University, 744 Motooka, Nishi-ku, Fukuoka, 819-0395, Japan

* To whom correspondence should be addressed. E-mail: t-mot@kanagawa-u.ac.jp.

Synthesis Procedure Details.

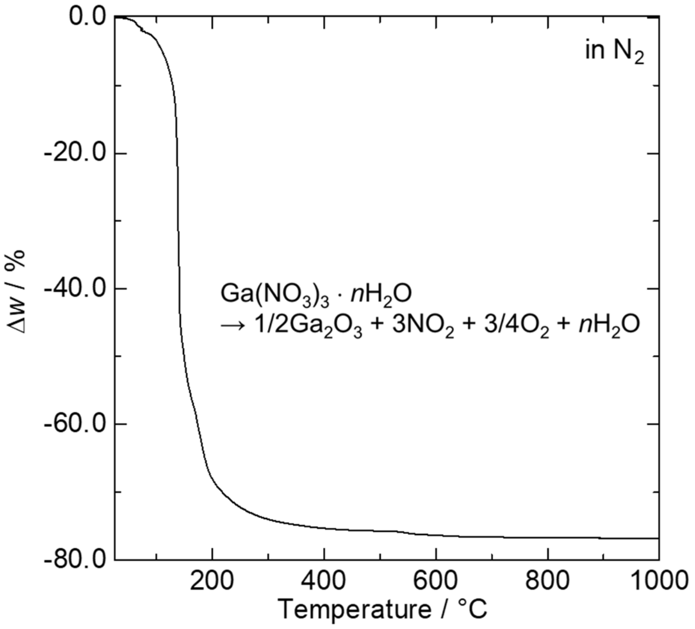

Figure S1. TG result of the $Ga(NO_3)_3{\cdot}nH_2O$ reagent used for sample synthesis.

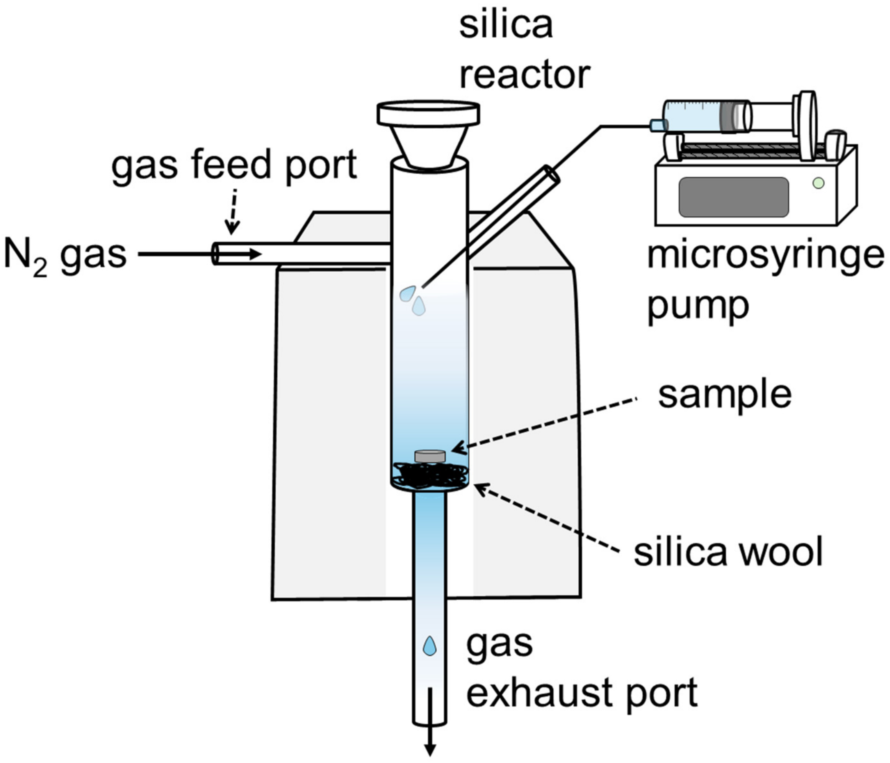

Figure S2. Schematic illustration of the custom-made reactor for "*vapor hydroxidation*."

Optimization of the reaction conditions for "*Vapor Hydroxidation*."

The Sr-Ga oxide precursor was heat-treated under various firing conditions in highly concentrated water vapor ("*vapor hydroxidation*"). XRD measurements of the resulting samples are presented in Figure S3. While samples synthesized in 30 – 50 vol% water vapor consistently contain hydroxides and hydrates, those prepared under 80 – 100 vol% water vapor exhibit the Sr-Ga oxy-hydroxide as the primary phase. Among the samples heat-treated in an atmosphere of 80 vol% water vapor, those treated at 500 and 700 °C are nearly phase-pure. These results indicate that the combination of reaction temperature and water vapor concentration is critical for enhancing the purity of the target Sr-Ga oxy-hydroxide. The optimal synthesis condition is presumed to be at 500 °C in 80 vol% water vapor.

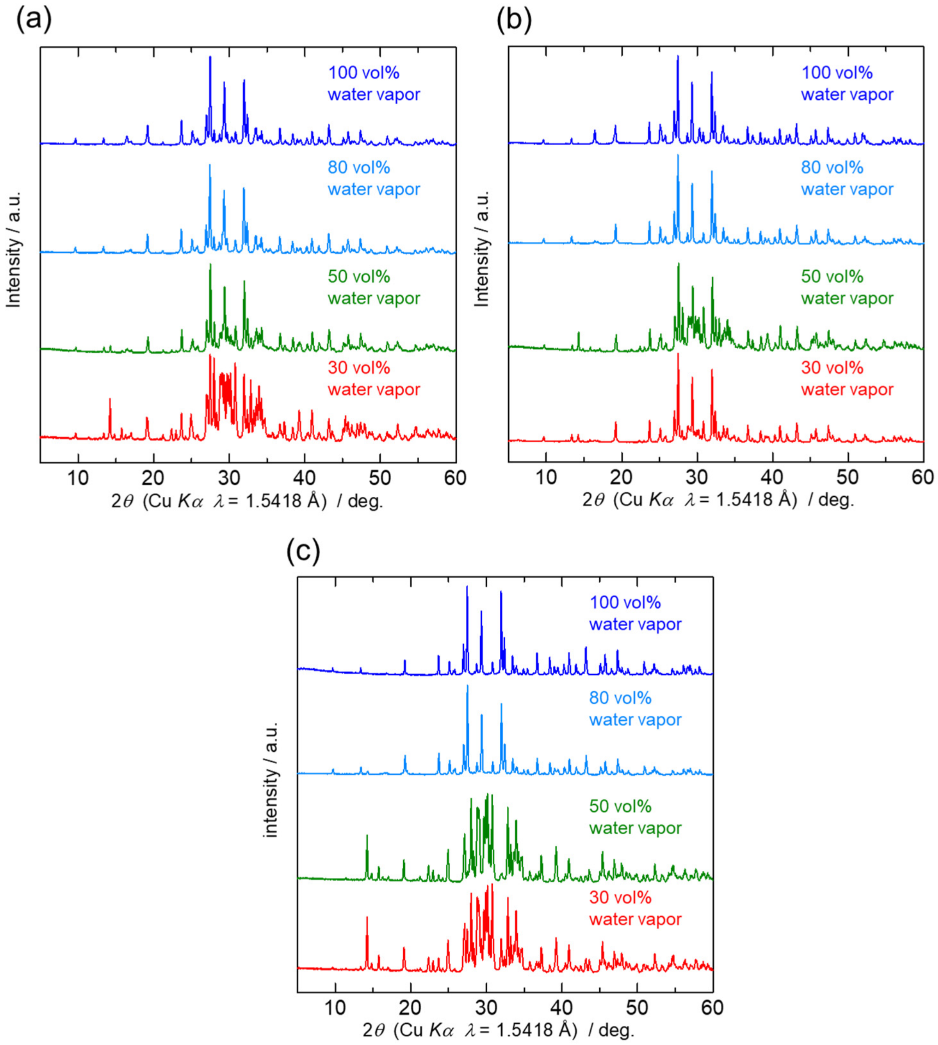

Figure S3. XRD patterns for the samples heat-treated under various conditions. (a) 300 °C, (b) 500 °C, and (c) 700 °C.

$Sr_2Ga_3O_6(OH)$ sample post-annealed at 600 °C for an extended duration

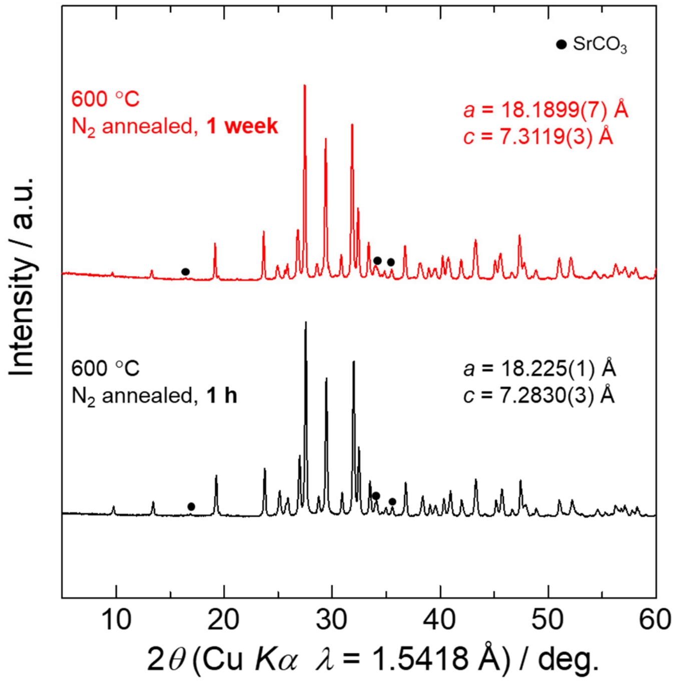

Figure S4. XRD patterns for $Sr_2Ga_3O_6(OH)$ samples post-annealed in flowing N_2 gas at 600 °C for one hour (black) and one week (red).

Sample Synthesis via Hydrothermal/Solvothermal Methods.

Syntheses of the Sr-Ga oxy-hydroxide were examined using conventional hydrothermal/solvothermal syntheses. $Sr(NO_3)_2$ (99.5 %, Kanto Chemical) and $Ga(NO_3)_3 \cdot nH_2O$ (99.999 %, Kojundo Chemical Laboratory; the *n* value was determined to be 6.82 by thermogravimetry), and NH_3 aq. (28%, FUJIFILM Wako Pure Chemical) were used as the starting materials. The synthesis conditions are detailed in Table S1. In each experiment, the reagent mixture was placed in a 50 mL pressure vessel (SAN-AI Kagaku Co.Ltd., HU-50) with a SUS304 outer vessel and a PTFE liner, and heat-treated at 200 °C for 20 hours.

Table S1. Summary of products from hydrothermal/solvothermal syntheses.

Raw materials	Solvent, Additive	Treatment temperature/time	Products
$Sr(NO_3)_2$ 2 mmol<br>$Ga(NO_3)_3 \cdot nH_2O$ 3 mmol	Water 20 mL	200 °C/20 h	GaO(OH)
$Sr(NO_3)_2$ 2 mmol<br>$Ga(NO_3)_3 \cdot nH_2O$ 3 mmol	Water 19mL<br>28 % NH_3 aq 1 mL	200 °C/20 h	GaO(OH)
$Sr(NO_3)_2$ 2 mmol<br>$Ga(NO_3)_3 \cdot nH_2O$ 3 mmol	Ethanol 19mL<br>28 % NH_3 aq 1 mL	200 °C/20 h	$Sr(NO_3)_2$<br>$(Ga_2O_3)_{1.07}$

X-ray powder diffraction patterns for the hydrothermal/solvothermal products are shown in Figure S5. These methods yielded GaO(OH) and $Sr(NO_3)_2$ as the primary phases, with no evidence of the target Sr-Ga oxy-hydroxide. Both hydrothermal and solvothermal syntheses proceeded via reactions in solution, suggesting that the Sr-Ga oxy-hydroxide does not form through liquid-phase processes. Notably, the "*vapor hydroxidation*" method is conducted at temperatures exceeding 500 °C, whereas the hydrothermal/solvothermal reactions typically occur at 100 – 200 °C, indicating that the formation of the Sr-Ga oxy-hydroxide requires higher reaction temperatures.

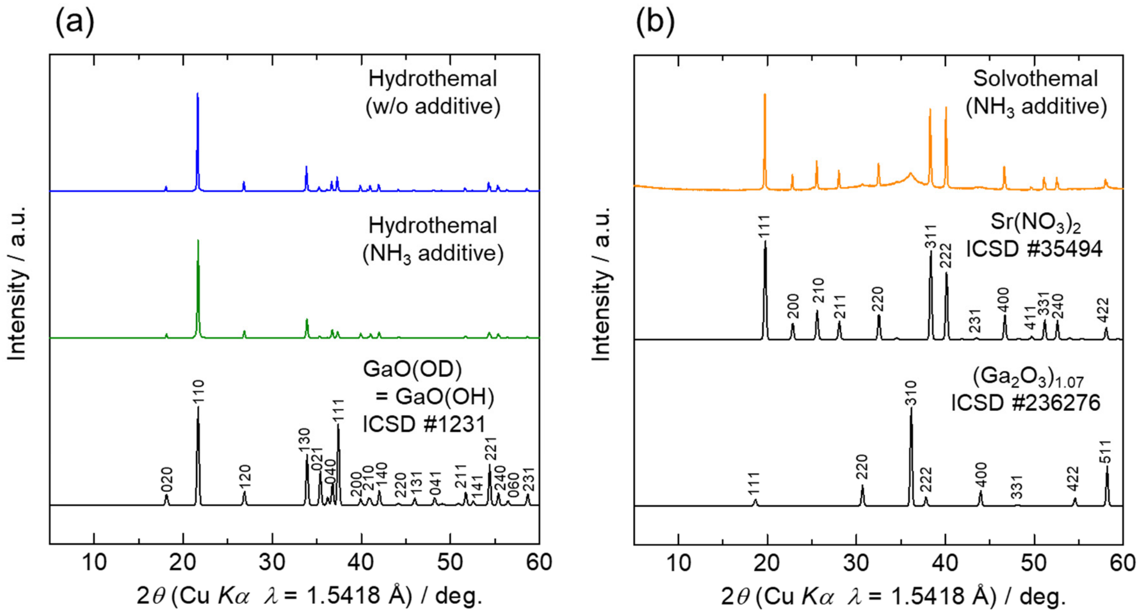

Figure S5. XRD patterns for the products of hydrothermal and solvothermal syntheses. (a) Hydrothermal product and (b) solvothermal product.

XRD data analysis.

Table S2. Parameters derived from Rietveld refinement of the XRD data. (a) Lattice parameters and R factors. (b) Structural parameters.

(a)

a / Å	c / Å	R_{wp} / %	R_p / %	S
18.226(1)	7.2830(3)	8.88	6.69	2.33

(b)

Atom	Site	x	y	z	Occ.	B_{iso}	BVS
Sr1	18*f*	0.2256(1)	0.4142(1)	0.0729(2)	1.000	1.00	1.82
Sr2	6*c*	0.00000	0.00000	0.2836(5)	1.000	1.00	3.06
Ga1	18*f*	0.5918(1)	0.4535(1)	0.2938(3)	1.000	1.00	2.96
Ga2	18*f*	0.6479(1)	0.0522(1)	0.1141(3)	1.000	1.00	3.98
O1	18*f*	0.5965(8)	0.5452(9)	0.4166(21)	1.000	2.00	-
O2	18*f*	0.1345(9)	0.1131(8)	0.1118(17)	1.000	2.00	-
O3	18*f*	0.5579(8)	0.0784(7)	0.0935(18)	1.000	2.00	-
O4	18*f*	0.3126(7)	-0.0549(7)	0.1527(15)	1.000	2.00	-
O5 / OH	18*f*	0.3395(15)	0.5642(12)	0.1920(27)	0.667	2.00	-

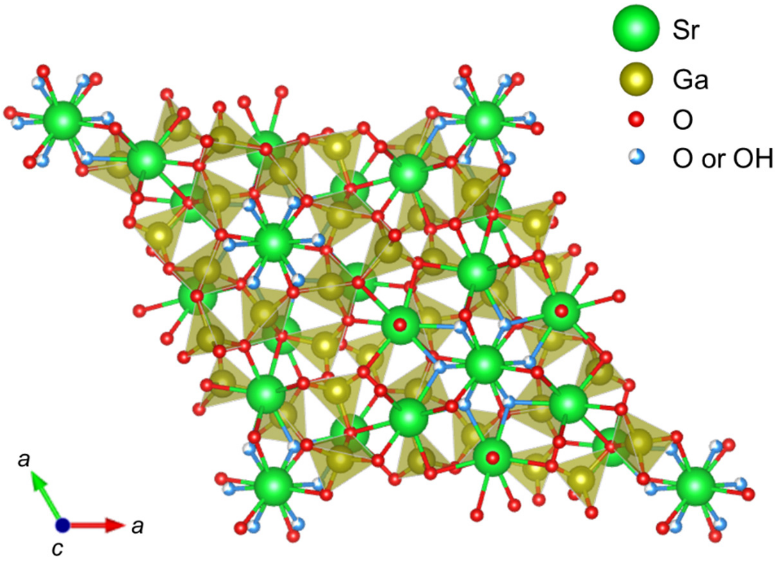

Figure S6. Crystal structure of the Sr-Ga oxy-hydroxide. The illustration was drawn using the VESTA software.[19]

ND data analysis.

(a)

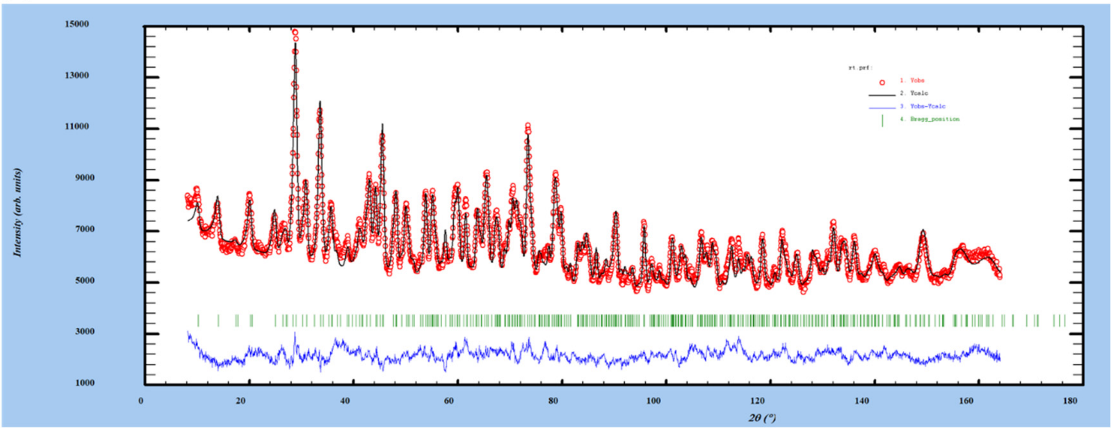

(b)

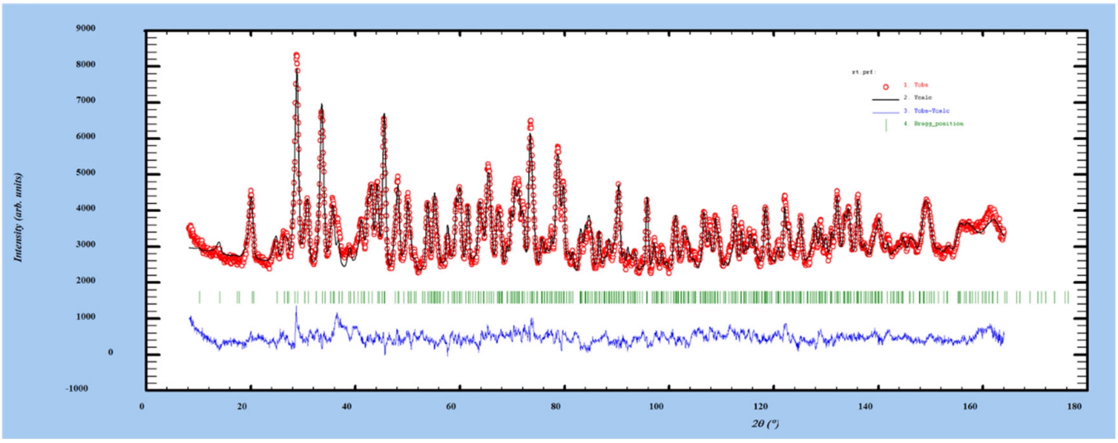

Figure S7. ND patterns for the Sr-Ga oxy-hydroxide measured at 3 K. (a) H sample and (b) D sample.

Table S3. Parameters derived from Rietveld refinement of the ND data. (a) Lattice parameters and *R* factors, (b) Structural parameters.

(a)

a / Å	c / Å	R_{wp} / %	R_p / %	χ^2
18.1904 (2)	7.2693 (1)	2.51	2.05	3.44

(b)

Atom	Site	x	y	z	Occ.	B_{iso}	BVS
Sr1	18*f*	0.2243(3)	0.4152(3)	0.0763(3)	1.000	1.573(75)	1.84
Sr2	6*c*	0.0000	0.0000	0.2756(12)	1.000	1.573(75)	2.51
Ga1	18*f*	0.5908(2)	0.4545(2)	0.2930(5)	1.000	0.027(40)	3.12
Ga2	18*f*	0.6454(2)	0.0506(2)	0.1119(5)	1.000	0.027(40)	2.99
O1	18*f*	0.5925(3)	0.5384(3)	0.4310(8)	1.000	0.856(44)	-
O2	18*f*	0.1412(4)	0.1142(3)	0.1175(7)	1.000	0.856(44)	-
O3	18*f*	0.5633(4)	0.0821(3)	0.1080(7)	1.000	0.856(44)	-
O4	18*f*	0.3200(3)	-0.0694(3)	0.1353(8)	1.000	0.856(44)	-
O5	18*f*	0.3372(5)	0.5694(5)	0.1848(10)	0.667	0.856(44)	-
H	18*f*	0.2986(13)	0.5260(15)	0.2790(3)	0.667	6.672(605)	-

Transmission electron microscope observation.

In the TEM image and selected-area electron diffraction (SAED) patterns shown in Figure S8, the grains are predominantly rhombohedral single crystals with sizes on the order of several hundred nanometers. The observed SAED pattern is consistent with the simulated patterns for an incident electron beam along the [0 0 −1] zone axis.

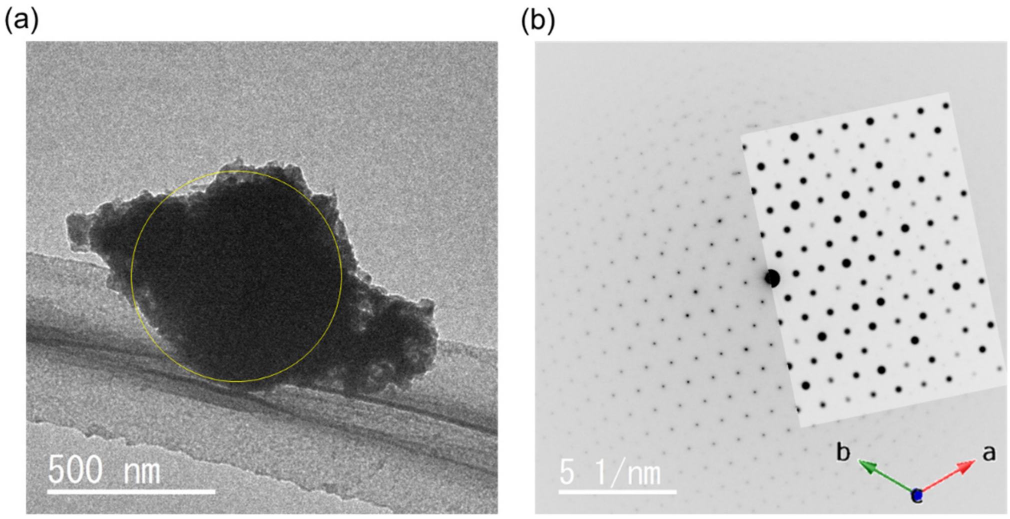

Figure S8. (a) TEM image and (b) Selected-area electron diffraction (SAED) pattern of $Sr_2Ga_3O_6(OH)$. The yellow circle indicates the area from which the diffraction pattern was selected.

Electrical conductivity.

For electrical conductivity measurements, the $Sr_2Ga_3O_6(OH)$ sample was pressed into pellets under a uniaxial pressure of approximately 5 MPa, followed by cold isostatic pressing at 250 MPa. The resulting compacts were sintered at 500 °C for 20 hours under approximately 80 vol% water vapor, and subsequently fired at 600 °C for 10 hours in flowing N_2 gas. The relative density of the sintered pellet was 79.7 %. A Pt paste (TR-7907, Tanaka Precious Metals) was applied to the sample surface and heat-treated at 600 °C for 1 hour under flowing N_2 gas. The bulk conductivity of the pellet was measured using an AC analyzer (SP-300, BioLogic) with an applied voltage of 30 mV and an AC frequency range of 7 MHz to 1 Hz. Measurements were conducted in a dry Ar atmosphere at 550 and 600 °C. The dew point of the outlet Ar gas from the measurement cell was monitored using a direct mirror-cooled dew point sensor (D-2-SR, General Eastern) and a dew point monitor (OptiSonde, General Eastern) to ensure that the water vapor partial pressure remained nearly constant below 0.25 hPa.

The bulk conductivity data of $Sr_2Ga_3O_6(OH)$ are presented in Figures S9 (a) ~ (c) and Table S4. The conductivity is as low as 10^{-7} S cm^{-1} at 600 °C, which is significantly lower than that of $[Ba_2O_x(OH)_y]_{0.55}InO_2$ ("*mf*-BI"), another oxy-hydroxide obtained via our "*vapor hydroxidation*" method. The poor conductivity of $Sr_2Ga_3O_6(OH)$ is likely attributed to its crystallographic nature. The crystal structure of $Sr_2Ga_3O_6(OH)$ is characterized by strong proton confinement within a narrow region near the Sr2 site. In contrast, *mf*-BI is proposed to facilitate the formation of effective proton diffusion pathways within the barium hydroxide block, resulting in substantially higher electrical conductivity compared to $Sr_2Ga_3O_6(OH)$.

Table S4. Resistance components and constant phase element (CPE) parameters used for fitting the Nyquist plots of $Sr_2Ga_3O_6(OH)$.

dry / °C	*R*0	*R*1	CPE1-*T*	CPE1-*P*	*R*2	CPE2-*T*	CPE2-*P*
550	-5.44×10^{1}	8.38×10^{6}	8.79×10^{-12}	9.68×10^{-1}	5.13×10^{7}	2.33×10^{-11}	9.40×10^{-1}
600	-1.18×10^{3}	2.70×10^{6}	9.27×10^{-12}	9.74×10^{-1}	1.18×10^{7}	3.26×10^{-11}	9.09×10^{-1}

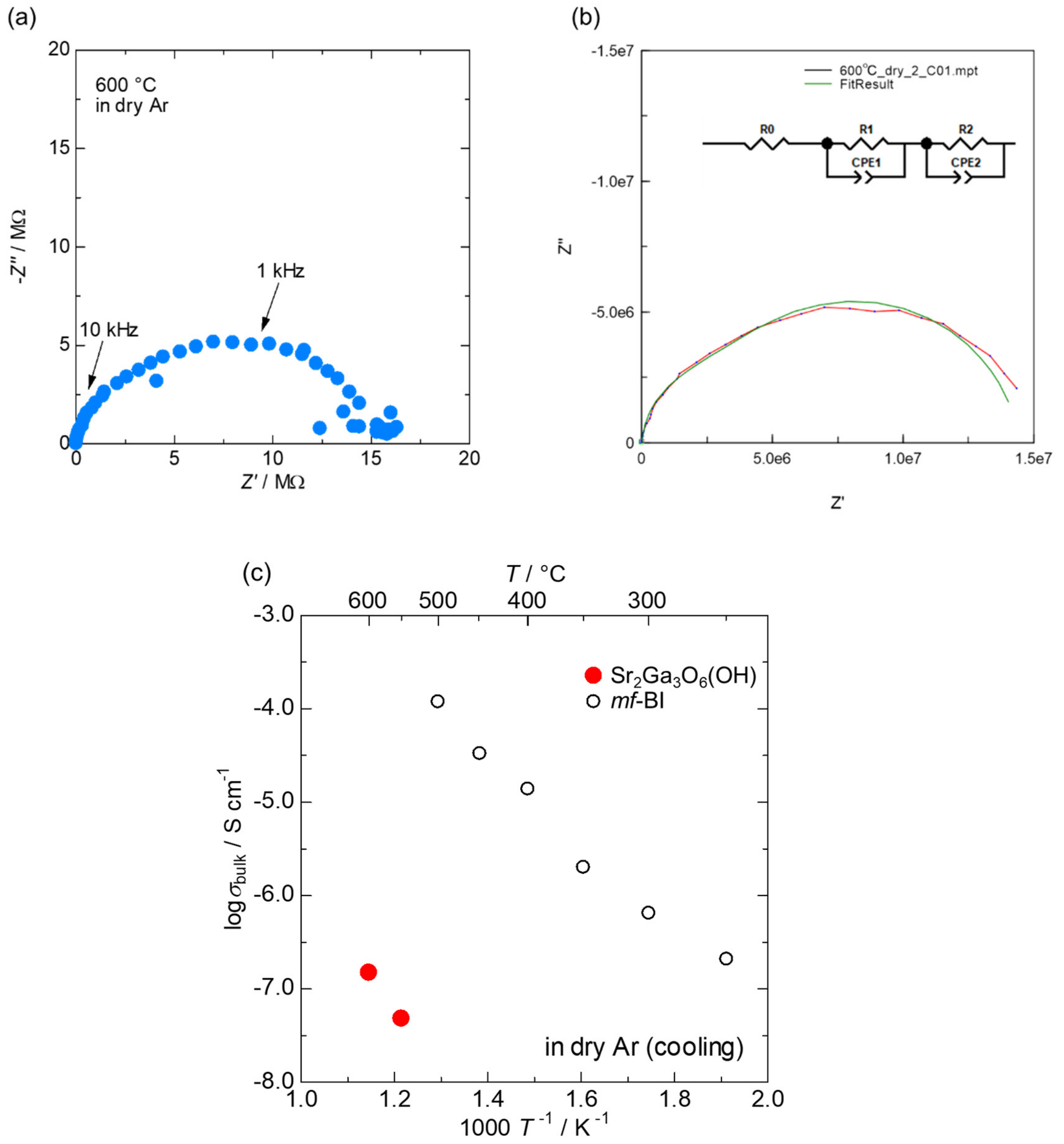

Figure S9. AC impedance data of $Sr_2Ga_3O_6(OH)$ in a dry Ar atmosphere. (a) Nyquist plot at 600 °C. (b) Deconvoluted nyquist plot at 600 °C. The inset shows a schematic representation of the equivalent circuit employed. (c) Arrhenius plots of bulk electrical conductivity upon cooling for $Sr_2Ga_3O_6(OH)$ and $[Ba_2O_x(OH)_y]_{0.55}InO_2$ ("*mf*-BI").

High-Temperature Synchrotron X-ray Diffraction (HT-SXRD) Study.

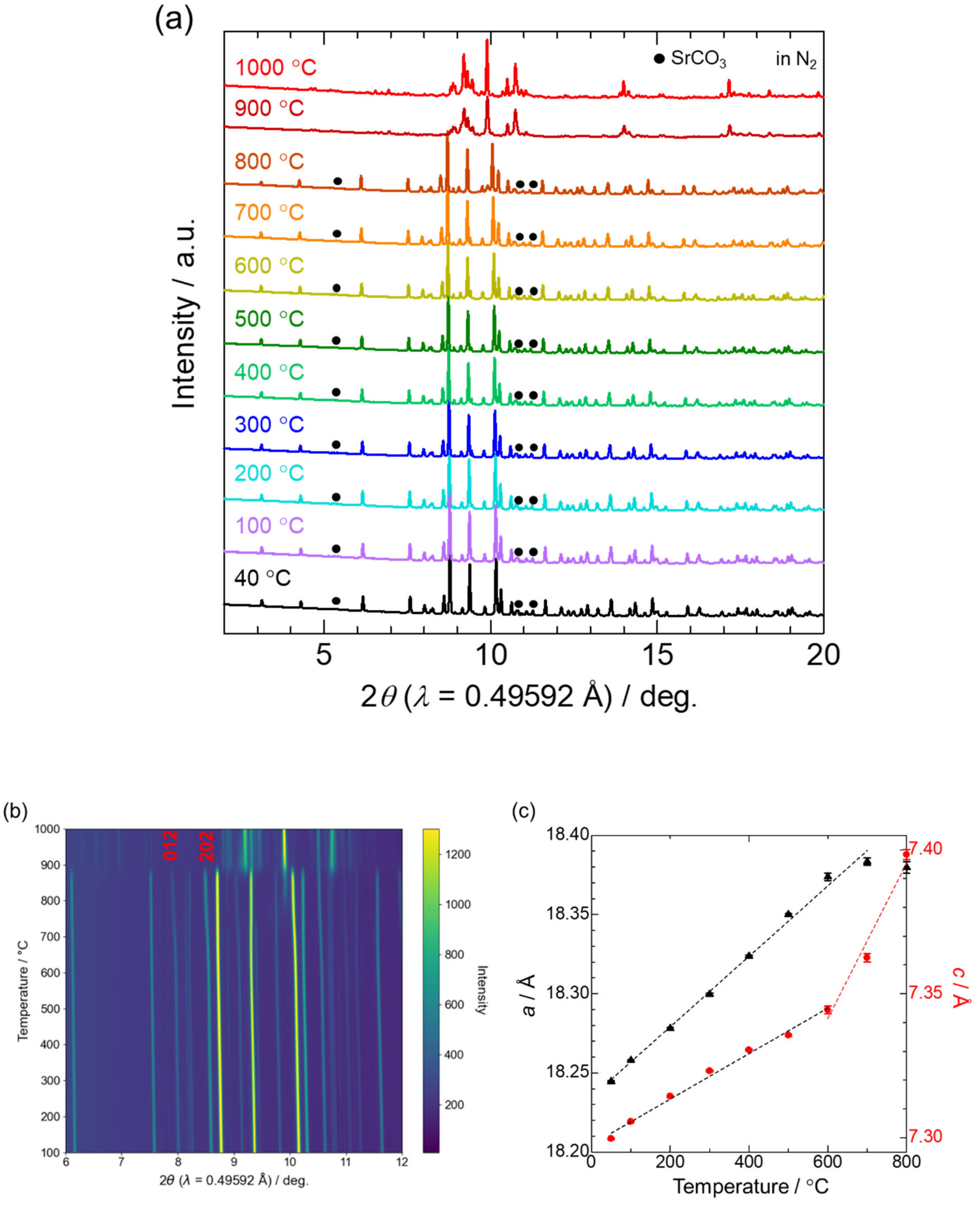

Figure S10. HT-SXRD patterns for $Sr_2Ga_3O_6(OH)$ up to 1000 °C. The diffraction data were collected using a wavelength of 0.49592 Å upon heating in flowing N_2 gas. (a) Diffraction patterns in the 2θ range of 2° ~ 20°. (b) Diffraction intensity map at 6° ~ 12°. (c) Lattice parameters as a function of temperature.

Thermal behaviors of (oxy-)hydroxides studied by thermogravimetry.

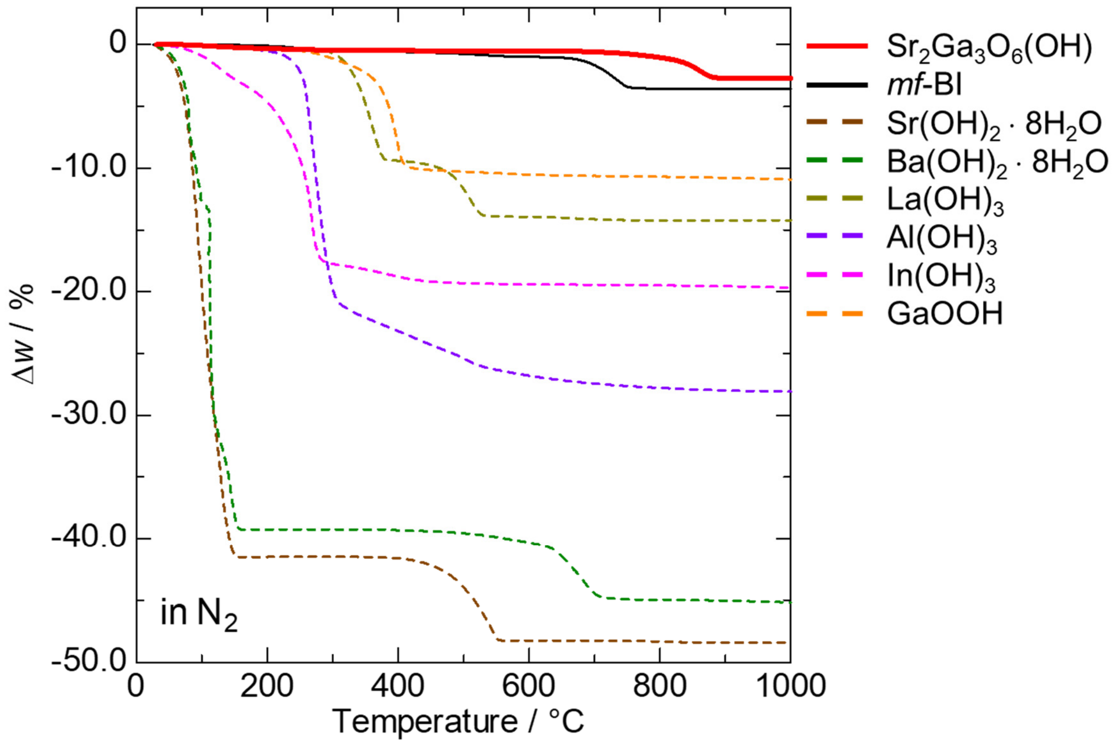

Figure S11. Summary of TG curves of various (oxy-)hydroxides. The data of $Sr_2Ga_3O_6(OH)$ and *mf*-BI are also presented.

In-situ FT-IR spectra of the $Sr_2Ga_3O_6(OH)$ and deuterium-substitited $Sr_2Ga_3O_6(OD)$.

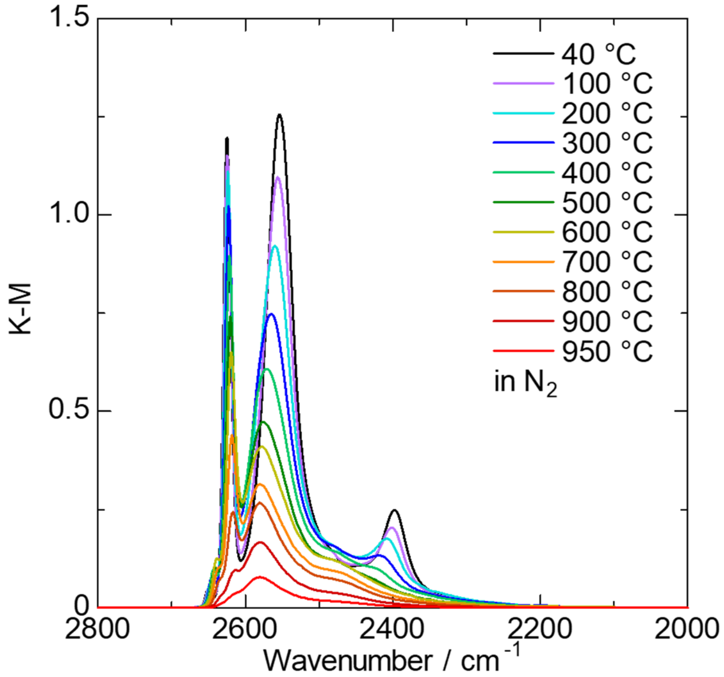

Figure S12. In-situ FT-IR spectra of the deuterium-substituted $Sr_2Ga_3O_6(OD)$ upon heating from 40 °C to 950 °C in flowing N_2 gas.

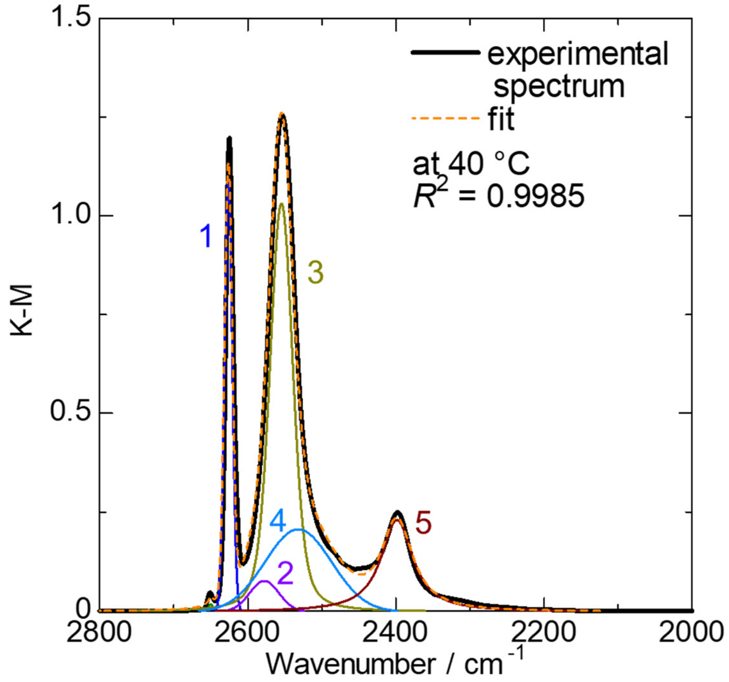

Figure S13. Deconvoluted FT-IR spectrum of $Sr_2Ga_3O_6(OD)$ at 40 °C.

Table S5. Peak positions and full widths at half maximum values for Peaks #1 – #5 in FT-IR spectra of $Sr_2Ga_3O_6(OH)$.

Peak	Position / cm^{-1}	Full wide at half maximum (wG) / cm^{-1}	Full wide at half maximum (wL) / cm^{-1}
1	3557	17.9	3.1
2	3508	62.2	0
3	3453	48.6	36.2
4	3411	150.0	0
5	3240	55.9	193.2

Table S6. Peak positions and full widths at half maximum values for Peaks #1 – #5 in FT-IR spectra of Deuterium-substituted $Sr_2Ga_3O_6(OD)$.

Peak	Position / cm^{-1}	Full wide at half maximum (wG) / cm^{-1}	Full wide at half maximum (wL) / cm^{-1}
1	2626	12.1	0
2	2586	45.8	1.9
3	2555	29.3	12.5
4	2530	105.8	0
5	2400	9.0	50.2

In-situ FT-IR spectra of $Sr_3Ga_2(OH)_{12}$, a reference compound.

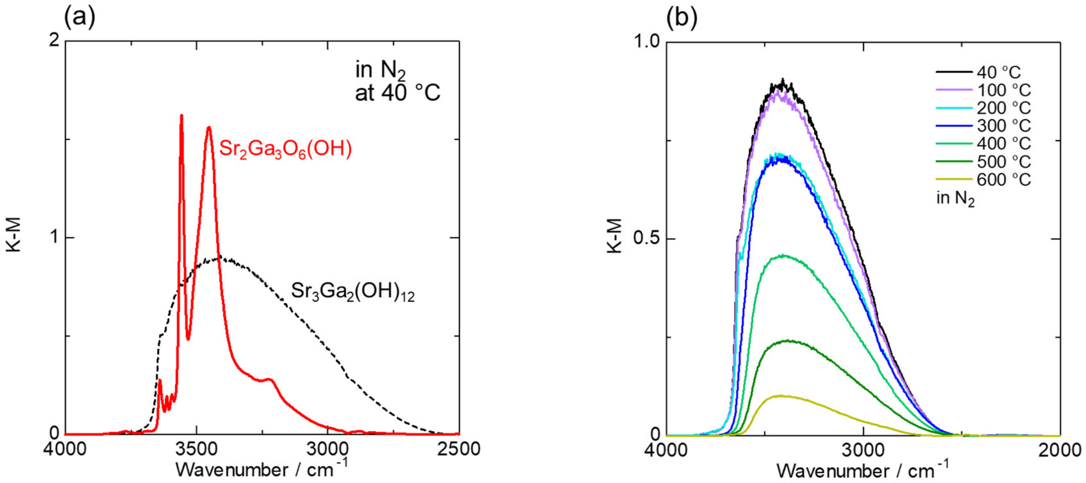

Figure S14. FT-IR spectra of $Sr_3Ga_2(OH)_{12}$. (a) Comparison of the FT-IR spectra (40 °C) between $Sr_3Ga_2(OH)_{12}$ and $Sr_2Ga_3O_6(OH)$. (b) In-situ FT-IR spectra of $Sr_3Ga_2(OH)_{12}$ upon heating from 40 °C to 600 °C in flowing N_2 gas.

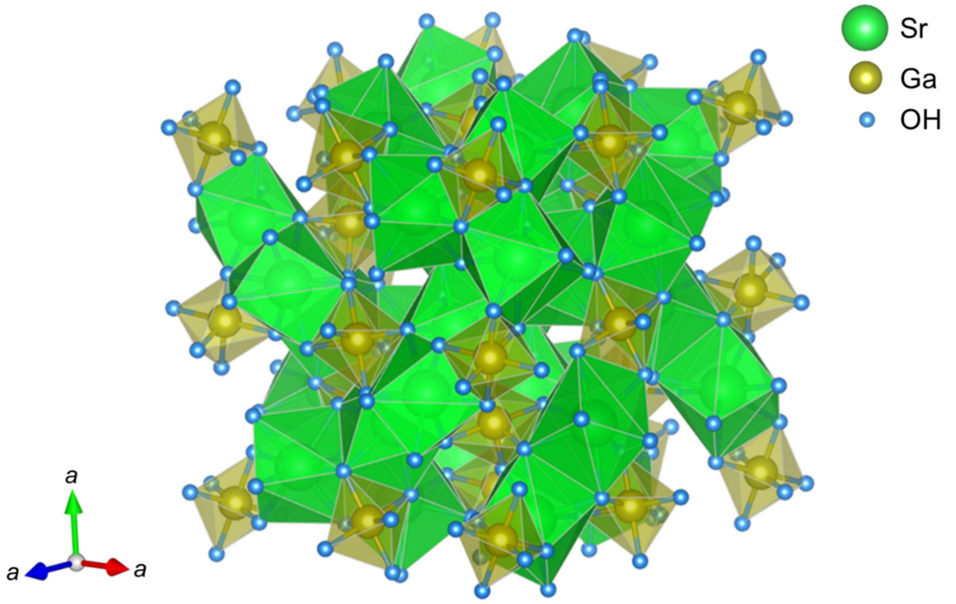

Figure S15. Crystal structure of $Sr_3Ga_2(OH)_{12}$. The illustration was drawn based on the structural model reported in (Ref. [51]; ICSD #249850).

References

(19) Momma, K.; Izumi, F. VESTA 3 for Three-Dimensional Visualization of Crystal, Volumetric and Morphology Data. *J Appl Crystallogr* **2011**, *44* (6), 1272–1276. https://doi.org/10.1107/S0021889811038970.

(51) Kim, M. K.; Jo, V.; Shim, I. W.; Ok, K. M. New Inorganic Helical Chain: Synthesis, Structure, Characterization, and Interconversion of $BaGa_2O_2(OH)_4$. *Inorg Chem* **2009**, *48* (4), 1275–1277. https://doi.org/10.1021/ic802187w.
